# Identification of Exosome-Related Genes Associated with Prognosis and Immune Infiltration Features in Head-Neck Squamous Cell Carcinoma

**DOI:** 10.3390/biom13060958

**Published:** 2023-06-07

**Authors:** Yuanhe You, Zhong Du, Guisong Xu, Zhuowei Tian, Meng Xiao, Yanan Wang

**Affiliations:** 1 Department of Oral and Maxillofacial-Head and Neck Oncology, Shanghai Ninth People’s Hospital, Shanghai Jiao Tong University School of Medicine, Shanghai 200011, China; youyuanhe@sjtu.edu.cn (Y.Y.); 821001@sh9hospital.org.cn (Z.D.);; 2 College of Stomatology, Shanghai Jiao Tong University, Shanghai 200011, China; 3 National Center for Stomatology, National Clinical Research Center for Oral Disease, Shanghai 200011, China; 4 Shanghai Key Laboratory of Stomatology, Shanghai 200011, China

**Keywords:** head and neck squamous cell cancer, exosome, prognosis, immune infiltration, bioinformatics

## Abstract

The highly immunosuppressive nature of head–neck squamous cell cancer (HNSCC) is not fully understood. Exosomes play crucial roles in the communication between cancer and non-cancer cells, but the clinical significance of the expression of exosome-related genes (ERGs) remains unclear in HNSCC. This study aimed to establish an HNSCC-ERGs model by using mass spectrometry (MS)-based label-free quantitative proteomics in combination with the TCGA primary HNSCC dataset. The study managed to classify the HNSCC patients into two subtypes based on the expression level of prognostic ERGs, which showed significant differences in prognosis and immune infiltration. LASSO regression algorithm was used to establish a risk prediction model based on seven risky genes (PYGL, ACTN2, TSPAN15, EXT2, PLAU, ITGA5), and the high-risk group was associated with poor survival prognosis and suppressive immune status. HPRT1 and PYGL were found to be independent prognostic factors through univariate and multivariate Cox regression analyses. Immune and ssGSEA analysis revealed that HPRT1 and PYGL were significantly related to immunosuppression, immune response, and critical signaling transduction pathways in HNSCC. Immunohistochemistry results further validated the expression level, clinical value, and immunosuppressive function of HPRT1 and PYGL in HNSCC patients. In conclusion, this study established molecular subtypes and a prediction risk model based on the ERGs. Furthermore, the findings suggested that HPRT1 and PYGL might play critical roles in reshaping the tumor microenvironment.

## 1. Introduction

The tumor microenvironment (TME) is a crucial factor in tumor progression and response to immunotherapy [1,2]. Targeting the related biological process may represent a promising approach to improving anti-tumor therapy, as suggested by Herrin [3]. Despite recent improvements in the treatment of head and neck squamous cell carcinoma (HNSCC), this disease remains highly lethal, with an overall 5-year survival rate of approximately 50% [4]. Immunotherapy has shown limited efficacy, benefiting only 15% of HNSCC patients, highlighting the need for a deeper understanding of the immunosuppressive mechanisms involved [5]. Although there have been some studies investigating the immune evasion mechanisms of cancer cells, our understanding of the maintenance of an immunosuppressive microenvironment remains incomplete and requires further investigation.

Exosomes, which are extracellular vehicles (EVs) measuring between 30 and 150 nm, play a crucial role in the communication between cancer and non-cancer cells in the TME [6]. An increasing body of evidence indicates that exosomes derived from cancer cells are essential regulatory factors in various tumorigenesis processes, such as tumor invasion, metastasis, and TME reprogramming to an immunosuppressive status [3,7,8]. In our previous study, we demonstrated that exosome-based communication was a critical contributor to macrophage activation, which could promote malignant biological properties via the IL6/Stat3/THBS1 feedback loop in HNSCC [9,10]. What’s more, exosomes play vital roles in T cell function. In metastatic melanoma, tumor cells greatly secrete exosomes expressing PD-L1, which participate in immune escape [11]. Exosomes present in the TME can inhibit the infiltration of activated CD8+ T cells, resulting in insensitivity to anti-PDL1 therapy [12]. An abundance of evidence supports the idea that targeting cellular communication mediated by exosomes may provide a potential strategy to enhance the responsiveness of tumor patients to immunotherapy regimens [13,14,15]. However, the role of exosome-related genes (ERGs) in HNSCC remains poorly understood.

Given the crucial roles of exosomes in reshaping the TME and their potential in antitumor processes, the present study aimed to construct a set of HNSCC exosome-related genes (ERGs) using data from The Cancer Genome Atlas (TCGA) database and genes encoding exosome proteins in HNSCC cell lines detected by mass spectrometry. A systematic study was performed to determine the gene signature of HNSCC ERGs by reclassifying HNSCC into two molecular subtypes based on ERG expression and investigating their prognostic value and distinct tumor infiltration patterns. The ultimate goal was to identify key exosome-related genes to facilitate further studies.

## 2. Materials and Methods

### 2.1. Differently Expressed Genes of HNSCC Samples

The study extracted genes expression data and related clinical information from the TCGA database (https://Portal.gdc.cancer.gov/repository, accessed on 12 April 2022). The analysis involved a total of 44 normal HNSCC samples and 504 HNSCC samples. The “Limma” package was utilized to identify differentially expressed genes (DEGs), with a threshold of adjusted *p*-value < 0.05 and |log2 (fold change)| > 1 set for the DEGs.

### 2.2. Isolation of HNSCC Exosome-Related Genes

HNSCC cell lines including SCC9, SCC25, and CAL27 were cultured in DMEM supplemented with 10% FBS, penicillin (100 U/mL), and streptomycin (100 μM/mL) at 37 °C in the presence of 5% carbon dioxide. Conditioned medium (CM) from the cells was obtained by washing the cells at up to 80% confluence and then culturing them for an additional 24 h with fresh DMEM (without FBS) [16]. The exosomes were extracted and identified as previous reported (Figure S1) [9,10].

### 2.3. Mass Spectrometry (MS)-Based Label-Free Quantitative Proteomics

HNSCC exosomes were subjected to label-free quantitative proteomics analysis using mass spectrometry. The samples were prepared and analyzed by Beijing BangFei Bioscience Co., Ltd. [17]. Mass spectrometry was performed using an Orbitrap Fusion mass spectrometer (Thermo Scientific, Waltham, MA, USA), and the eluted peptides from each sample were analyzed. The acquired data were processed using Proteome Discoverer software, and the uniprot-human_160701.fasta database was used as the reference. Gene symbols encoding HNSCC exosome proteins were obtained from the Genecards database [18]. To identify genes shared among the HNSCC cell lines, Venn analysis was performed.

### 2.4. Enrichment Analysis and ssGSEA Analysis

Herein, we conducted a comprehensive analysis of the biological functions and signaling pathways of the candidate genes using Gene Ontology (GO) [19] and Kyoto Encyclopedia of Genes and Genomes (KEGG) [20] enrichment analyses. In addition, we utilized the R packages “ClusterProfiler” and “GSVA,” employing the ssGSEA method and Spearman correlations to compute several functional pathways.

### 2.5. Identification of Prognostic HNSCC Exosome-Related Genes

In order to ascertain the prognostic significance of the HNSCC exosome-related genes, we utilized Cox regression analysis to assess the associations between the expression of each gene and the survival outcomes based on the TCGA-HNSCC cohort. We performed log-rank tests and summarized *p*-values and hazard ratios (HRs) with 95% confidence intervals (CIs). A gene was considered to have prognostic value if its *p*-value was less than 0.05, and we identified a set of 25 genes for further analysis.

### 2.6. Exploration of Gene Alteration Landscape of Prognostic ERGs

The cBio Cancer Genomics Portal (cBioPortal, https://www.cbioportal.org/, accessed on 20 September 2022) is a web portal for visual analysis of genomics in the TCGA database [21]. In this study, the cBioPortal platform was used to analyze the the effects of genetic alterations in the 25 identified prognostic ERGs on the prognosis of HNSCC patients, in terms of overall survival (OS) and disease-specific survival (DSS). The statistical significance of the results was assessed with a *p*-value threshold of <0.05.

### 2.7. Construction of Molecular Subgroups Using Consensus Clustering

The study utilized the “ConsensusClusterPlus” package to cluster the 25 prognostic ERGs genes into different subtypes in 504 HNSCC samples obtained from the TCGA database. The optimal k value was used to split the TCGA-HNSCC cohort into two clusters: cluster 1 (ERGs low expression group) and cluster 2 (ERGs high expression group). Principal component analysis (PCA) was conducted to discern the differences between the two patient groups. The survival difference between clusters 1 and 2 was evaluated using Kaplan–Meier plots.

### 2.8. Immune Infiltration Analysis

In order to evaluate the immune infiltration results across different subgroups, we utilized the “immuneeconv” R software package [22], which encompasses a variety of state-of-the-art algorithms such as TIMER [23], MCPCOUNTER [24], CIBERSORT [25], EPIC [26], and QUANTISEQ [27]. The immune analysis outcomes were visualized through the use of R packages “ggplot2” and “pheatmap.”

### 2.9. Construction of Risk Model and Nomograms Based on Prognostic HNSCC ERGs

The current study aimed to investigate the potential association between the expression of HNSCC ERGs and prognosis in HNSCC patients through univariate Cox regression analysis (*p* < 0.05). Furthermore, a risk model was developed for HNSCC ERGs using the least absolute shrinkage and selection operator (LASSO) regression algorithm. The m6A/m1A/m5C-regulated genes were utilized to calculate the risk score (risk score = S (ExpmRNAn × βmRNAn)) [28]. The high- and low-risk groups were then compared using Kaplan–Meier curves and *p*-values were generated using log-rank tests. A forest plot was employed to display the *p*-value, HR, and 95% CI of each variable using the “forestplot“ R package. Finally, a nomogram was created based on the results of multivariate Cox proportional hazards analysis of the HNSCC ERGs.

### 2.10. Immunohistochemistry (IHC) Staining

The IHC staining protocol was executed with meticulous adherence to our previously reported methodology [9]. Post-deparaffinization, rehydration, and antigen retrieval steps were performed, followed by quenching of endogenous peroxidase activity. Protein expression was determined using primary antibodies (rabbit anti-human HPRT1 antibody (#15059-1-AP), rabbit anti-human PYGL antibody (#15851-1-AP), and mouse anti-human CD8 antibody, all of which were acquired from Proteintech (Wuhan, China). To assess the extent of immunoreactivity, the immunoreaction score (IRS) was computed by multiplying the percentage of positive cells and the staining intensity, as previously reported [29].

## 3. Results

### 3.1. Identification of HNSCC Exosome-Related Genes

**Figure 1:**
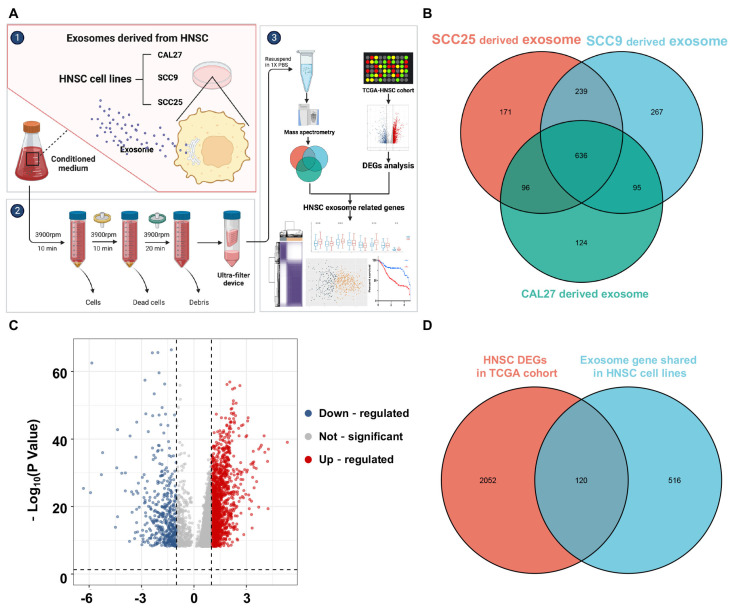
Identification of HNSCC exosome—related genes. (**A**) Workflow of identifying HNSCC exosome—related genes. (**B**) Venn diagram of HNSCC exosome proteins shared in HNSCC cell lines (CAL27, SCC9, SCC25) measured by mass spectrometry-based quantitative proteomics. (**C**) Volcano plot of differentially expressed genes between tumor and adjacent normal tissues in TCGA cohort. (**D**) Venn diagram showing 120 overlapping genes among genes encoding HNSCC exosome proteins and differentially expressed genes in HNSCC from the TCGA.

Exosomes have emerged as significant regulators of intercellular communication. The present study aimed to establish a set of exosome-related genes in HNSCC. Exosomes were isolated from the cell culture media of three HNSCC cell lines, namely CAL27, SCC9, and SCC25. These genes were then overlapped with the differentially expressed genes (DEGs) of the TCGA-HNSCC cohort for further analysis (Figure 1A). A total of 636 proteins were identified that were shared in exosomes derived from the CAL27, SCC9, and SCC25 cell lines (Figure 1B). Consequently, the genes encoding these proteins were matched using the National Center for Biotechnology Information (NCBI) database. DEGs were identified from the TCGA database at a level of fold change > |2| and *p*-value < 0.05, leading to the identification of 2172 genes (Figure 1C, Table S1). A Venn diagram was used to determine the intersection between the exosome genes from the HNSCC cell lines and DEGs from the TCGA-HNSCC cohort, which revealed 120 overlapping genes (Figure 1D). The expression levels of these 120 HNSCC ERGs are presented in Table S2.

### 3.2. Functional Enrichment Analysis and Determination of Prognostic HNSCC ERGs

**Figure 2:**
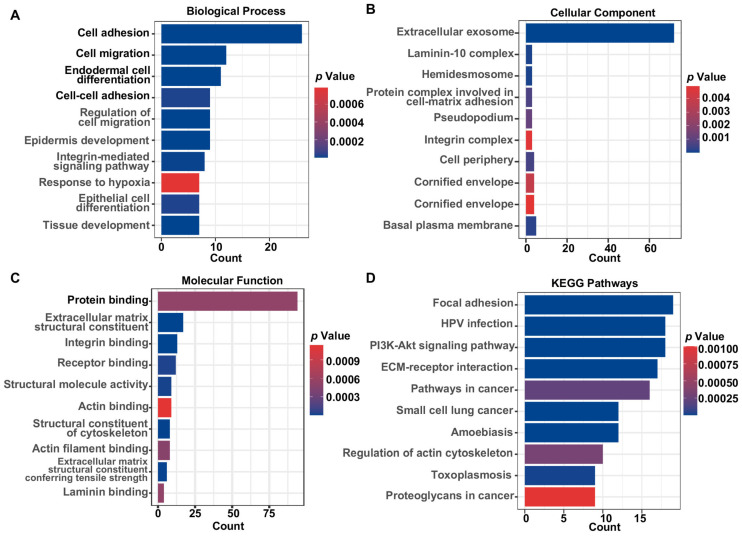
Functional enrichment analysis of exosome-related genes in HNSCC. Bar plot graph for GO and KEGG pathway enrichment. (**A**) Biological process. (**B**) Cellular component. (**C**) Molecular function. (**D**) KEGG pathways. Bar length represents gene count and color represents the *p*-value.

To ascertain the functions of the identified HNSCC ERGs, a comprehensive analysis was carried out via Gene Ontology (GO) and KEGG functional enrichment analyses. The GO analysis results showed that the 120 HNSCC ERGs were primarily implicated in cellular adhesion, migration, differentiation of epidermal/epithelial cells, response to hypoxia, extracellular exosome, and protein binding (Figure 2A–C). In parallel, the KEGG pathway analysis results indicated that the 120 HNSCC ERGs were prominently associated with focal adhesion, HPV infection, the PI3K/AKT pathway, ECM-receptor interaction, and pathways in cancer (Figure 2D).

**Figure 3:**
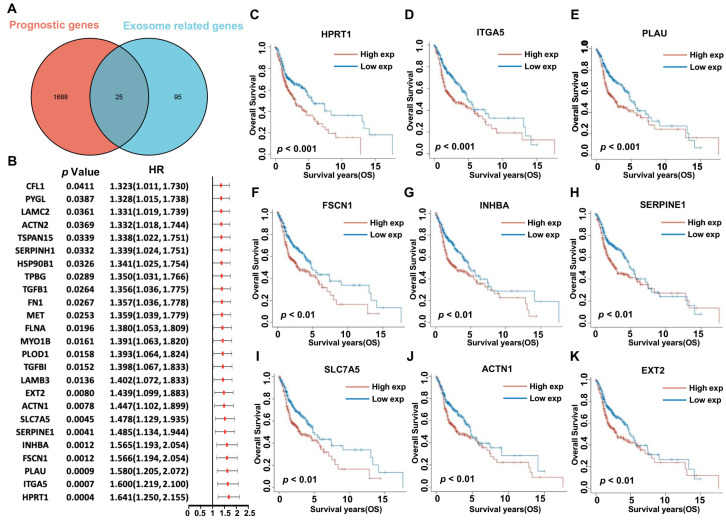
The prognostic value of exosome genes in HNSCC. (**A**) Venn diagram showing 25 prognostic exosome-related genes in HNSCC cohort. (**B**) Univariate Cox regression analysis of OS for each exosome-related gene with *p* < 0.05. Overall survival curves of top 9 prognostic exosome-related genes, (**C**) HPRT1, (**D**) ITGA5, (**E**) PLAU, (**F**) FSCN1, (**G**) INHBA, (**H**) SERPINE1, (**I**) SLC7A5, (**J**) ACTN1, and (**K**) EXT2, in HNSCC patients between the high-/low-expression groups.

To identify HNSCC ERGs with prognostic implications, the TCGA-HNSCC cohort was subjected to univariate Cox regression analysis, which revealed 1713 genes as potential prognostic markers. Subsequently, 25 genes were identified as prognostic HNSCC ERGs associated with increased risk, with HRs > 1 (Figure 3A,B). The prognostic value of these HNSCC ERGs was further validated by Kaplan–Meier survival curves in high-/low-expression groups of HNSCC patients. The representative Kaplan–Meier survival curves were obtained for 9 genes (Figure 3C–K).

### 3.3. Expression Profiles of 25 Prognostic HNSCC ERGs

**Figure 4:**
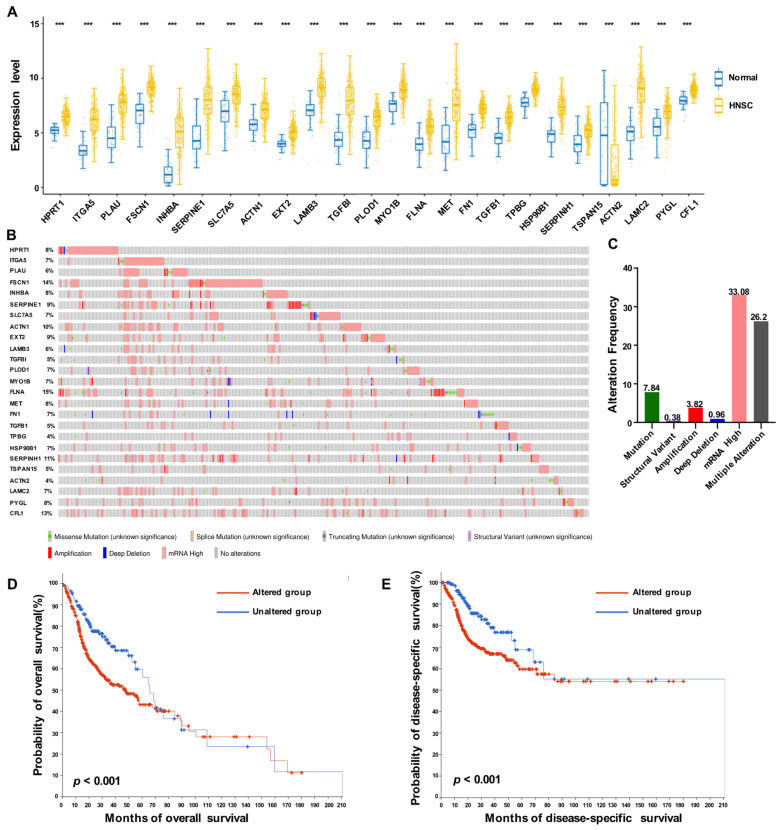
Expression profile of ERGs in HNSCC. (**A**) The mRNA expression levels of 25 prognostic ERGs in the TCGA-HNSCC cohort. (**B**,**C**) Gene alterations of 25 prognostic ERGs were analyzed via cBioPortal in HNSCC. (**D,E**) Kaplan–Meier plots comparing overall survival and disease-specific survival in cases with/without 25 ERGs alterations. *** *p* < 0.001.

Based on the fold change analysis, we observed that most of the ERGs were upregulated in HNSCC tissues relative to normal tissues, except for ACTN2, which was downregulated (Figure 4A). To further understand the genetic alterations of HNSCC ERGs, we utilized the cBioPortal website and found that 72.28% of the 523 HNSCC cases had ERG alterations (Figure 4B). The genetic alterations included mutation (7.84%), structural variant (0.38%), amplification (3.82%), deep deletion (0.96%), mRNA high (33.08%), and multiple alterations (26.2%) (Figure 4C). Our analysis revealed that these genetic alterations significantly affected the overall survival (Figure 4D, *p* < 0.001) and disease-specific survival (Figure 4E, *p* < 0. 001) of HNSCC patients. Additionally, we performed Spearman’s correlation analysis to analyze the correlations among the 25 prognostic ERGs, and the results are shown in Figure S2.

### 3.4. Molecular Subtype of HNSCC Based on 25 Prognostic ERGs

**Figure 5:**
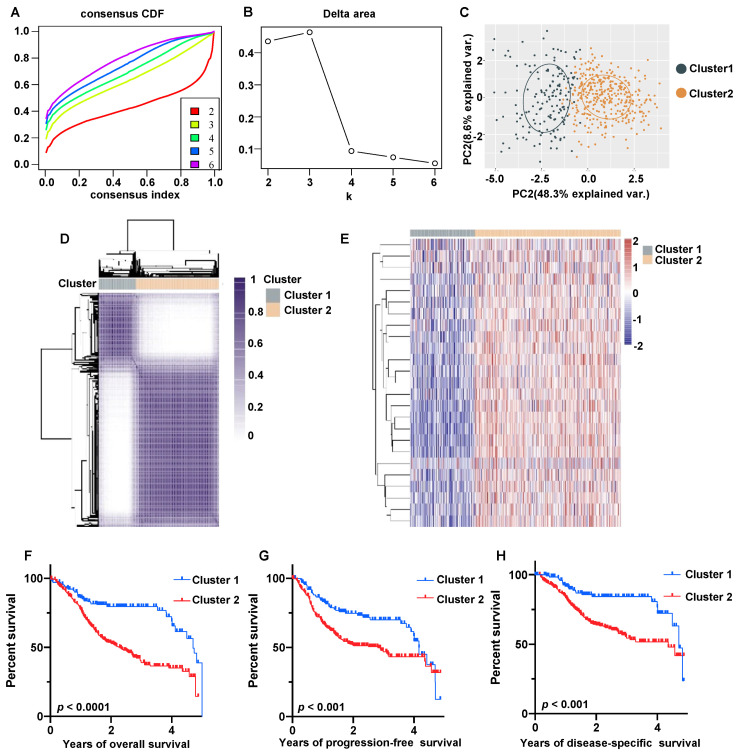
Molecular subtype of HNSCC based on 25 prognostic ERGs. (**A**–**C**) The consensus clustering of 25 prognostic ERGs in HNSCC; (**A**) Cumulative distribution function curve, (**B**) delta area curve, (**C**) PCA. (**D**) HNSCC patients (*n* = 504) were grouped into the ERG−low subtype (cluster 1, *n* = 156) and ERG-high subtype (cluster 2, *n* = 348) according to the consensus clustering matrix (k = 2). (**E**) Heatmap of ERG expression in different subtypes of HNSCC. (**F**–**H**) Kaplan–Meier plots comparing overall survival (**F**), progression-free survival (**G**), and disease−specific survival (**H**) between clusters 1 and 2.

To examine the association between the expression levels of HNSCC exosome-related genes and molecular subtypes of HNSCC, we conducted a consensus clustering analysis using data from the TCGA cohort comprising 504 HNSCC patients (Figure 5A–C). Herein, we managed to identify an optimal k value of 2, and the HNSCC patients were divided into two clusters: cluster 1 with low ERG expression and cluster 2 with high ERG expression (Figure 5D). The expression patterns of ERGs across different HNSCC subtypes are shown in Figure 5E. Notably, cluster 1 demonstrated higher overall survival (Figure 5F), progression-free survival (Figure 5G) and disease-specific survival (Figure 5H) rates than cluster 2, and the differences were statistically significant (*p* < 0.001). Additionally, significant differences were observed between the two clusters in terms of lymph node metastasis and grade stage (*p* < 0.05, Table 1). The differential expression analysis revealed that cluster 2 had 522 upregulated genes and 82 downregulated genes relative to cluster 1 (Figure S3A,B). KEGG pathway analysis indicated that cluster 2 was associated with activation of the TGF-β signaling pathway, regulation of actin cytoskeleton, focal adhesion, the PI3K-Akt signaling pathway, ECM receptor interaction, and suppression of tyrosine metabolism, tight junction, steroid biosynthesis, glycolysis, and the estrogen signaling pathway (Figure S3C). The Gene Ontology analysis showed that the upregulated genes were involved in extracellular matrix organization/disassembly, endodermal cell differentiation, collagen metabolic process, and cell-substrate adhesion (Figure S3D).

### 3.5. HNSCC ERG-High Subtype Was Associated with Immunosuppressive TME

**Figure 6:**
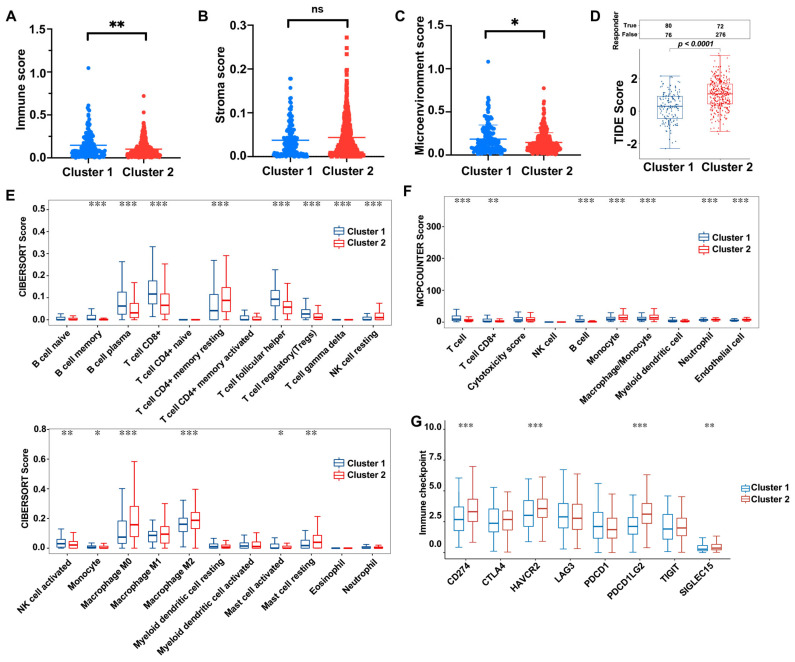
Association between ERG-high subtype and the immunosuppressive tumor microenvironment. (**A**–**C**) Immune score (**A**), stroma score (**B**), and microenvironment score (**C**) were calculated by ESTIMATE algorithm. (**D**) TIDE scores of clusters 1 and 2 were analyzed using the TIDE algorithm to predict tumor immunotherapy response rate. (**E**,**F**) CIBERSORT (**E**) and MCPCOUNTER (**F**) scores revealed differences in immune cell infiltration between clusters 1 and 2. (**G**) Differential expression levels of immune checkpoint-associated genes in clusters 1 and 2. * *p* < 0.05, ** *p* < 0.01, *** *p* < 0.001, ns: not significant.

Exosomes play a crucial role in regulating intercellular communication. Here, we investigated the characteristics of immune infiltration in the tumor microenvironment (TME) of HNSCC. Our analysis revealed a significant difference between the ERG-high cluster (cluster 2) and ERG-low cluster (cluster 1) in terms of low immune score (Figure 6A) and microenvironment score (Figure 6C) but not stroma score (Figure 6B). We utilized the TIDE algorithm to predict the tumor immune response rate and found that cluster 2 had a significantly higher TIDE score than cluster 1 (*p* = 1.4 × 10^−15^), suggesting that patients in the ERG-low group might benefit more from immune checkpoint inhibitor therapy (ICBs) (Figure 6D). Using the CIBERSORT algorithm, we analyzed immune heterogeneity between the two molecular subgroups (Figure 6E). The results showed that cluster 2 was negatively associated with B cell plasma, CD8+ T cells, activated CD4+ memory T cells, and activated NK cells (*p* < 0.001) but positively associated with resting CD4+ memory T cells and resting NK cells (*p* < 0.001). Further validation using the MCPCOUNTER algorithm showed that cluster 2 was associated with stronger immunosuppressive status, with a positive relationship with T cells, CD8+ T cells, and B cells (*p* < 0.01) (Figure 6F). Similar results were obtained using the EPIC algorithm (Figure S4A), QUANTISEQ algorithm (Figure S4B), and TIMER algorithm (Figure S4C). We also analyzed the expression levels of immune checkpoint markers in both HNSCC subtypes and found that the expression of CD274, HAVCR2, PDCD1LG2, and SIGLEC15 was elevated in cluster 2 (Figure 6G). Overall, our findings suggested that HNSC patients in the ERG-high subtype might have a greater likelihood of developing an immunosuppressive microenvironment, which could ultimately contribute to poor prognosis.

### 3.6. Construction of 25 Prognostic HNSCC ERGs Risk Model

**Figure 7:**
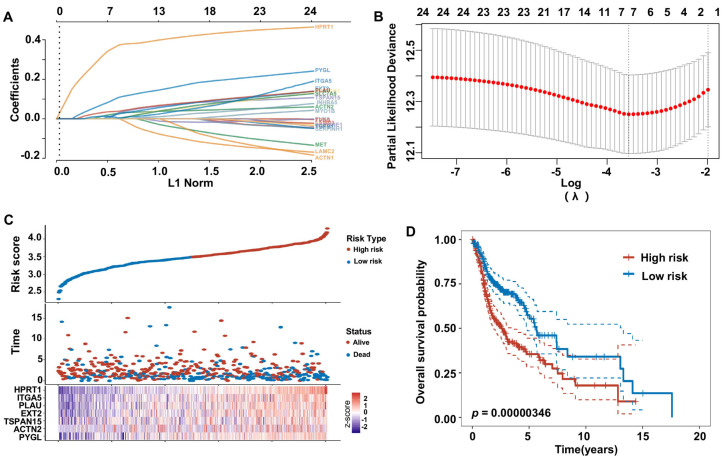
A novel risk model constructed using the LASSO method to predict the prognosis of HNSCC patients based on ERGs. (**A**) Coefficients of 25 prognostic ERGs represented by λ parameter. (**B**) LASSO COX regression model was used to draw the partial likelihood deviance versus log(λ). (**C**) The correlations between risk scores and ERGs. (**D**) Curves of overall survival for high−and low− risk groups of HNSCC patients.

To further investigate the association between ERGs and prognosis in HNSCC, we employed the least absolute shrinkage and selection operator (LASSO) Cox regression algorithm to determine the minimum λ value (Figure 7A,B). Subsequently, we constructed a 7-gene signature based on the optimal λ value. The correlation between the expression levels of these 7 genes and the risk score is displayed in Figure 7C. The risk score was calculated as follows: Riskscore = (0.0944) × PYGL + (0.0237) × ACTN2 + (0.0133) × TSPAN15 + (0.0161) × EXT2 + (0.0368) × PLAU + (0.0092) × ITGA5 + (0.3567) × HPRT1. Based on the risk score, HNSCC patients were stratified into two groups, and Kaplan–Meier survival analysis indicated that patients in the high-risk group had a worse prognosis than those in the low-risk group (Figure 7D).

### 3.7. Correlation between Immune Score and Risky ERGs in HNSCC

**Figure 8:**
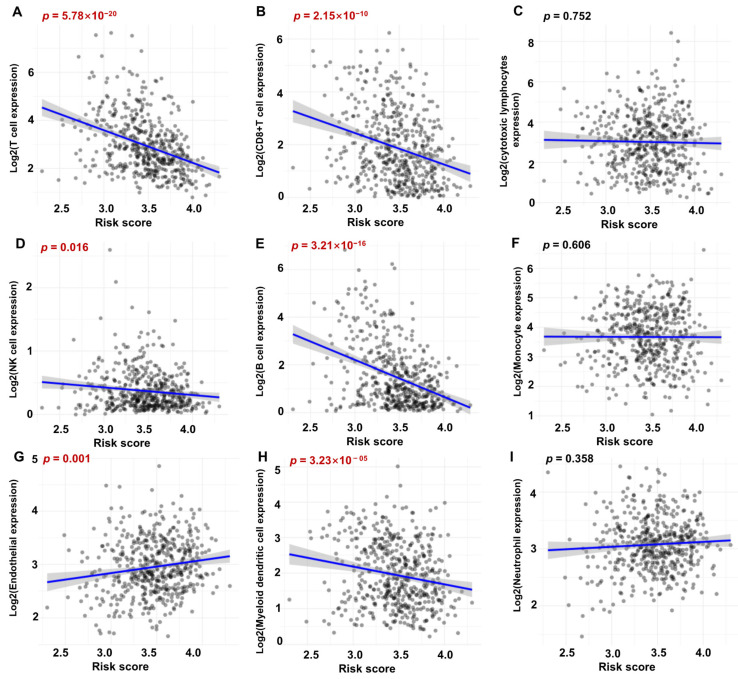
The correlation between the risk model and immune score in HNSCC via MCPCOUNTER algorithm. (**A**) T cells. (**B**) CD8+ T cells. (**C**) Cytotoxic lymphocytes. (**D**) NK cells. (**E**) B cells. (**F**) Monocytes/macrophages. (**G**) Endothelial cells. (**H**) Myeloid dendritic cells. (**I**) Neutrophils.

**Figure 9:**
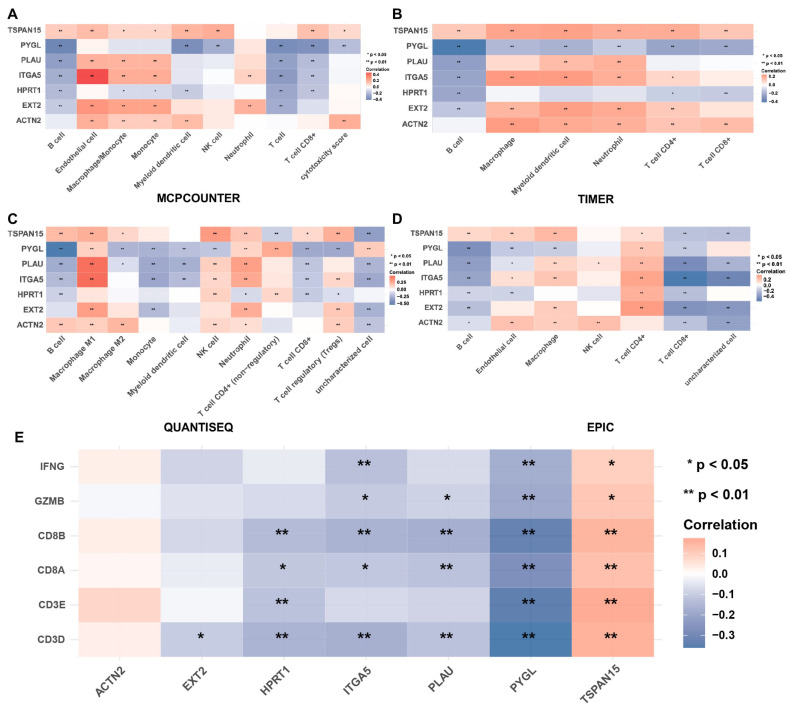
The correlation between the 7 risky genes and immune cell infiltration. Four algorithms were adopted for analysis in HNSC, (**A**) MCPCOUNTER, (**B**) TIMER, (**C**) QUANTISEQ, and (**D**) EPIC. (**E**) The correlation between the 7 risky genes and CD8+ T cell markers. * *p* < 0.05, ** *p* < 0.01.

To assess the potential relationship between the 7-gene risk model and immune score in HNSCC, we utilized the MCPCOUNTER (Figure 8) and TIMER algorithms (Figure S5) for analysis. Our results showed that the risk score was significantly and negatively associated with the infiltration of T cells (Figure 8A), CD8+ T cells (Figure 8B), NK cells (Figure 8D), B cells (Figure 8E), and myeloid dendritic cells (Figure 8H) in the tumor microenvironment, while no significant differences were found for the level of cytotoxic lymphocytes (Figure 8C), monocytes (Figure 8F), and neutrophils (Figure 8I). Interestingly, the risk score was positively correlated with endothelial cells, suggesting that HNSCC—derived exosomes might contribute to angiogenesis (Figure 8G). We further evaluated the correlation between the expression level of the 7 risky ERGs and immune cell infiltration. We used 4 algorithms (MCPCOUNTER, TIMER, QUANTISEQ, and EPIC) for analysis in HNSCC and found that the expression levels of HPRT1 and PYGL were related to decreased CD8+ T cell infiltration level across all four algorithms (Figure 9A–D). We also examined the correlation between the expression profile of the 7 risky genes and CD8+ T cell markers (CD3D, CD3E, CD8A, CD8B, IFNG, GZMB) and found that EXT2, HPRT1, ITGA5, PYGL, and PLAU were negatively associated with CD8+ T cell abundance. The correlation between the 7 risky genes and other infiltration cell markers was also explored (Figure S6). These results suggested that the 7-gene risk model might be associated with immune cell infiltration in HNSCC, and the identified risky genes might play a potential role in modulating the immune response in HNSCC.

### 3.8. A prognostic Nomogram Based on the 7 Risky ERGs

**Figure 10:**
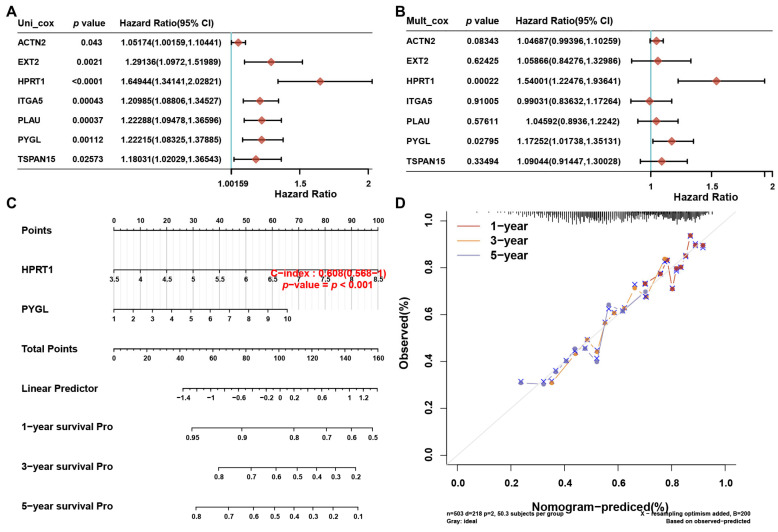
Prognostic nomogram constructed based on the 7 risky ERGs in HNSCC patients. (**A**,**B**) Hazard ratio and *p*-value of the constituents involved in univariate (**A**) and multivariate (**B**) Cox regression analyses of seven prognostic ERGs in HNSCC. (**C**) Nomogram to predict the overall survival rate of HNSCC patients. (**D**) Calibration curve for the overall survival nomogram model. The dashed diagonal line represents the ideal nomogram.

In our study, we developed a prognostic nomogram that utilized the expression levels of the 7 risky genes to predict the survival probability in HNSCC patients. Univariate (Figure 10A) and multivariate (Figure 10B) Cox analyses were conducted to assess the relationship between individual genes and patient prognosis. The results showed that HPRT1 and PYGL might be independent prognostic factors in HNSCC. Based on these findings, a nomogram was constructed that incorporated the expression levels of HPRT1 and PYGL (Figure 10C). The predictive nomogram was able to accurately predict the 1-, 3-, and 5-year overall survival rates in the entire cohort (Figure 10D). Together with the immune analysis presented in Figure 9, our results suggested that HPRT1 and PYGL might be potential key ERGs in HNSCC.

### 3.9. Genetic Function Analysis of HPRT1 and PYGL

**Figure 11:**
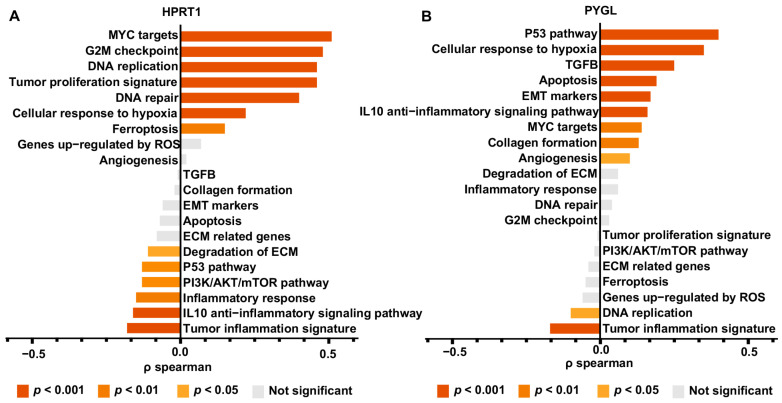
The correlation between core ERGs and 19 pathways using the ssGSEA algorithm, (**A**) HPRT1, (**B**) PYGL.

The present study explored the GEPIA database to investigate the expression levels and functions of HPRT1 and PYGL in multiple cancer types. HPRT1 expression was found to be upregulated in several cancer types, including BRCA, CESC, COAD, DLBC, ESCA, HNSC, LUAD, LUSC, PAAD, READ, SKCM, STAD, THYM, and UCEC, while it was downregulated in GBM, LAML, LGG, and TGCT (Figure S7A). Similarly, PYGL expression was upregulated in GBM, HNSC, KIRC, KIRP, LAML, LGG, PAAD, SKCM, and TGCT, but it was downregulated in ACC, DLBC, KICH, and THYM (Figure S7B). Furthermore, gene set enrichment analysis (GSEA) revealed that HPRT1 was positively associated with MYC targets, G2M checkpoint, tumor proliferation signature, DNA repair, cellular response to hypoxia, and ferroptosis, while it was negatively associated with ECM degradation, the P53 pathway, inflammatory response, and the IL10 anti-inflammatory signaling pathway (Figure 11B). In contrast, PYGL was positively correlated with the P53 pathway, cellular response to hypoxia, TGFβ, apoptosis, EMT marker, angiogenesis, collagen formation, and MYC targets, but it was negatively correlated with DNA replication and the tumor inflammation signature (Figure 11B). KEGG and GO functional enrichment analyses were also conducted, and the HPRT1 high expression group was associated with activation of the Wnt signaling pathway, drug metabolism, purine metabolism, ether lipid metabolism, skin development, epidermis development, and suppression of the IL17 signaling pathway compared to the HPRT1 low expression group (Figure S8). Similarly, the PYGL high expression group was highly associated with PI3K signaling, the Hippo pathway, human papillomavirus infection, extracellular matrix organization/assembly, and epidermis development. Overall, these findings suggested that HPRT1 and PYGL might play important roles in cancer development and prognosis (Figure S9).

### 3.10. Overexpression of HPRT1 and PYGL Were Correlated with Tumor Progression and CD8+ T Cell Infiltration in HNSCC Patients

**Figure 12:**
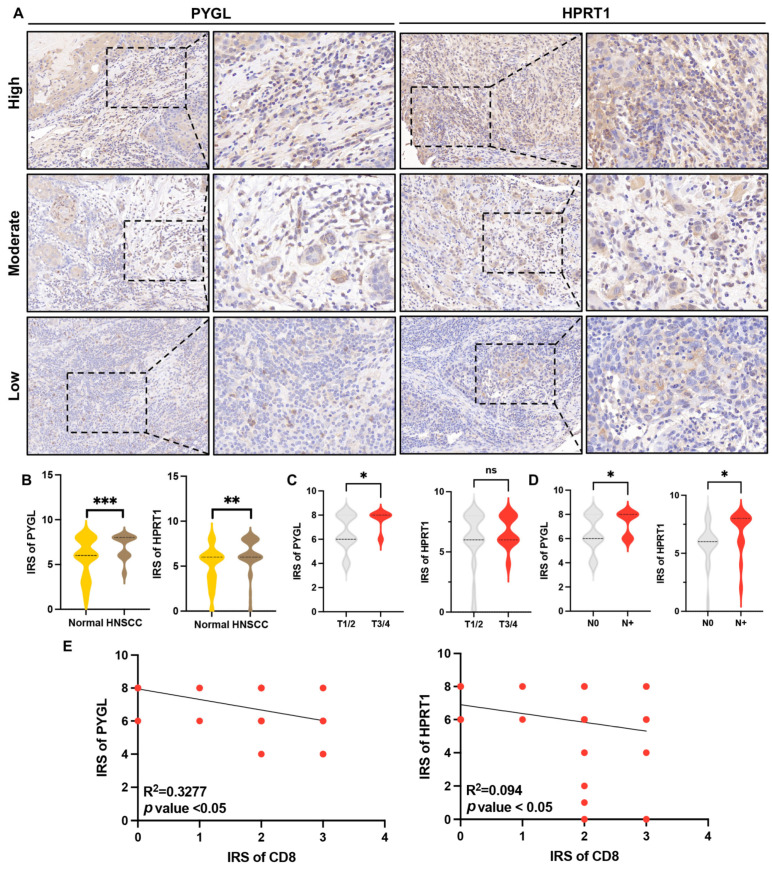
Overexpression of HPRT1 and PYGL correlated with tumor progression and decreased CD8+ T cell infiltration. (**A**) Representative images of high, moderate, and low expression of HPRT1 in HNSCC were obtained using a tissue microarray assay that included 48 paired samples (400×). (**B**) IHC scores of HPRT1 and PYGL in primary HNSCC cohort (*n* = 48). (**C**,**D**) The correlation between HPRT1 and PYGL expression, tumor size, and lymph node metastasis was analyzed in HNSCC patients. (**E**) Correlation between HPRT1, PYGL, and CD8 protein expression in the HNSCC cohort. * *p* < 0.05, ** *p* < 0.01, *** *p* < 0.001, ns: not significant.

In this study, the protein expression levels of HPRT1 and PYGL were evaluated using immunohistochemistry in a validated primary HNSCC cohort (*n* = 48). Representative images of low, moderate, or high levels based on IRS score are shown in Figure 12A. Significantly higher expression levels of HPRT1 and PYGL were observed in HNSCC tissue as compared to the paired normal control (*p* < 0.01) (Figure 12B). No statistical difference was observed based on age (Figure S10). Correlation analysis was performed to investigate the relationship between expression levels of PYGL and HPRT1, tumor size, and lymph node metastasis. The expression level of PYGL was found to be positively correlated with tumor size (Figure 12C, *p* < 0.05) and lymph node metastasis (Figure 12D, *p* < 0.05). However, only lymph node status was correlated with HPRT1 expression (Figure 12C,D). In addition, the study validated the relationship between HPRT1, PYGL, and CD8+ T cell infiltration level (Figure 8) in the primary HNSCC cohort. A negative correlation was observed between HPRT1 and PYGL expression levels and CD8+ T cell infiltration level (Figure 12E). These findings suggested that HPRT1 and PYGL might be associated with tumor progression and might induce an immunosuppressive microenvironment.

## 4. Discussion

Numerous studies have demonstrated the importance of exosomes in investigating the mechanisms underlying the development, advancement, metastasis, and immune evasion of HNSCC [30,31,32]. The complexity of the TME plays a critical role in tumor progression. In addition to cancer cells, the TME comprises a variety of non-tumor components, such as immune cells, endothelial cells, fibroblasts, and cellular metabolites. Exosomes are a specific type of EV with a wide range of biological functions in mediating cellular interactions between cancer and non-tumor cells through the proteins, nucleic acids, and metabolites they transport [33]. However, the precise roles of exosome-mediated gene regulation in the progression of HNSCC remain unclear. In this study, we aimed to investigate the biological functions and prognostic values of the HNSCC ERGs.

In this study, a HNSCC ERG set was constructed using mass spectrometry-based label-free quantitative proteomics and the TCGA-HNSCC dataset. Through differential analysis and functional enrichment analysis, 120 genes were identified as HNSCC ERGs. Among them, 25 genes were found to have a significant value in overall survival. All the included HNSCC patients were classified into two subtypes, and we observed that the ERG-high expression cluster was associated with poor prognosis and an immunosuppressive microenvironment. A risk model and nomogram were developed based on seven risky genes. The study also revealed that HPRT1 and PYGL were the core ERGs owing to their prognostic value and potential immunosuppressive function, which was further confirmed in the validated HNSCC cohort.

Exosomes play a crucial role in tumorigenesis as essential immunomodulators, particularly in remodeling the TME to induce tumor metastasis and an immunosuppressive status [34]. For example, exosomal PDL1 derived from tumor cells leads to immunosuppression and correlates with anti-PDL1 immunotherapy [11]. Exosomal CD73 derived from HNSCC tumor cells mediates phenotypic and functional changes in TAMs to induce immune tolerance [30]. However, systematic research on exosome-related genes in cancer based on big data is still lacking. In this study, we conducted a comprehensive analysis of ERGs in the HNSCC cohort. Identifying cancer subgroups based on gene expression is meaningful in clinical practice [35]. Based on the prognostic ERGs, we reclassified HNSCC patients into two subtypes, which showed significant clinical implications. A prognostic model was established based on PYGL, ACTN2, TSPAN15, EXT2, PLAU, ITGA5, and HPRT1. Univariate and multivariate Cox regression analyses further revealed that HPRT1 and PYGL might be independent factors for constructing a nomogram model.

HPRT1 was reported to play a critical role in cancer progression and chemoresistance, including HNSCC. Overexpression of PYGL also predicted poor prognosis in solid tumors. Both genes were detected in HNSCC-derived exosomes and validated in primary HNSCC tissue. In the immune infiltration analysis, both genes were significantly correlated with immune inhibition. We focused on T cells, which are the main anti-tumor immune cells. Accordingly, overexpression of HPRT1 and PYGL was negatively related to T cell (especially in CD8+ T cell) infiltration level and CD8+ T cell effectors (IFNG, GZMB). Herein, the two genes were identified as key ERGs of HNSCC. The immune surveillance system functions as a tumor suppressor in the early stage of tumor development. T cell metabolism altered by oncometabolite is a novel capability of cancer cells for immune evasion. Thus, we hypothesize that the two metabolic enzymes might reprogram T cell metabolism to sustain immune inhibition status. However, more research is needed to explore the potential mechanism.

HPRT1 is recognized as a significant contributor to cancer progression and chemoresistance, particularly in HNSCC [36]. PYGL overexpression is a predictor of poor prognosis in solid tumors [37]. HPRT1 and PYGL were detected in HNSCC exosomes and verified in primary HNSCC tissue. Upon conducting immune infiltration analysis, we found that both genes exhibited a significant correlation with immune inhibition, particularly with T cells, which are the main anti-tumor immune cells. Notably, overexpression of HPRT1 and PYGL was negatively correlated with the infiltration level of T cells, particularly CD8+ T cells, and CD8+ T cell effector molecules, including IFNG and GZMB. Therefore, we identified HPRT1 and PYGL as crucial ERGs in HNSCC. The immune surveillance system plays a crucial role in the early stages of tumor development as a tumor suppressor [38]. A novel cancer cell capability for immune evasion is altering T cell metabolism through oncometabolites [39]. Hence, we postulate that the two metabolic enzymes might reprogram T cell metabolism to sustain immune inhibition status. However, further research is required to investigate the potential mechanisms involved.

In summary, we developed a set of 120 HNSCC ERGs and identified a correlation between HNSCC reclassification, poor prognosis, and an immunosuppressive microenvironment. Additionally, we established a risk model and nomogram based on seven risky genes (PYGL, ACTN2, TSPAN15, EXT2, PLAU, ITGA5, HPRT1). Through immune infiltration analysis and determination of prognostic value, HPRT1 and PYGL were identified as key regulators carried by exosomes, based on their prognostic value and correlation with immune inhibition. However, further research is necessary to determine the mechanisms by which HPRT1 and PYGL induce immune evasion.

## Figures and Tables

**Table 1:** Clinical characteristics of clusters 1 and 2 based on exosome-related genes.

	Characteristics	Cluster 1	Cluster 2	*p*-Value
Age	Mean (SD)	61 (11.6)	61.1 (12)	
	Median [MIN, MAX]	61 [24, 87]	61 [19, 90]	0.969
Gender	Female	37	97	
	Male	119	251	0.386
Smoking	Non-smoking	41	72	
	Smoking	112	269	0.202
pT stage	T1	15	19	
	T2	49	97	
	T3	40	93	0.125
	T4	8	17	
	T4a	38	114	
	TX	6	5	
	T4b		3	
pN stage	N0	70	172	
	N1	21	60	
	N2	3	16	
	N2a	9	9	
	N2b	31	46	0.035
	N2c	11	30	
	N3	1	6	
	NX	10	9	
pM stage	M0	146	333	
	M1	1	4	
	MX	9	11	0.337
pTNM stage	I	8	17	
	II	23	58	
	III	33	58	0.621
	IVA	86	205	
	IVB	4	9	
	IVC	2	1	
Grade	G1	24	38	
	G2	78	223	
	G3	40	79	
	G4	2		
	GX	9	8	0.023
New tumor event type	Metastasis	10	9	
	Primary	3	6	
	Recurrence	11	28	0.188

## Data Availability

Further inquiries can be directed to the corresponding authors.

## Supplementary Materials

The following are available online at https://www.mdpi.com/article/10.3390/biom13060958/s1, Figure S1: The size and quantity of exosomes were measured using NTA; Figure S2: The correlation among 25 prognostic ERGs was assessed via Spearman’s correlation analysis; Figure S3: Differential expression genes and functional enrichment analysis of cluster1 and cluster2. (A) The volcano plot was constructed using the fold change > 2 and *p*-adjust < 0.05. (B) The top 50 up-regulated genes and top 50 down-regulated genes were showed in the heatmap. (C) Bubble graph for KEGG pathway enrichment. (D) Bubble graph for GO enrichment; Figure S4: Immune infiltration score of cluster 1 and cluster2 analyzed via four algorithms, EPIC (A), QUANTISEQ (B), TIMER (C); Figure S5: The relationship between the risk model and immune cell infiltration in HNSCC via TIMER algorithm; Figure S6: The association between the 7 risky genes and immune cell markers in HNSCC TME; Figure S7: Gene expression level of HPRT1 and PYGL in 33 kinds cancer. (A) HPRT1. (B) PYGL. Red: high expression, Green: low expression; Figure S8: Functional enrichment analysis between HPRT1 high expression group and HPRT1 low expression group in HNSCC. (A) KEGG pathway enrichment analysis. (B) GO functional enrichment analysis; Figure S9: Functional enrichment analysis between PYGL high expression group and PYGL low expression group in HNSCC. (A) KEGG pathway enrichment analysis. (B) GO functional enrichment analysis; Figure S10: The correlation between HPRT1, PYGL expression and age; Table S1: The DEGs in TCGA-HNSCC cohort; Table S2: The expression levels of these 120 HNSCC ERGs.
