# Supplementary material for: Identification of Exosome-Related Genes Associated with Prognosis and Immune Infiltration Features in Head-Neck Squamous Cell Carcinoma
Source: Biomolecules. 2023 Jun 7;13(6):958. doi: 10.3390/biom13060958 (PMC10296256; doi:10.3390/biom13060958)
Supplement: Supplementary file 1 [file biomolecules-13-00958-s001.zip › biomolecules-2303385-supplementary.pdf]

Figure S1

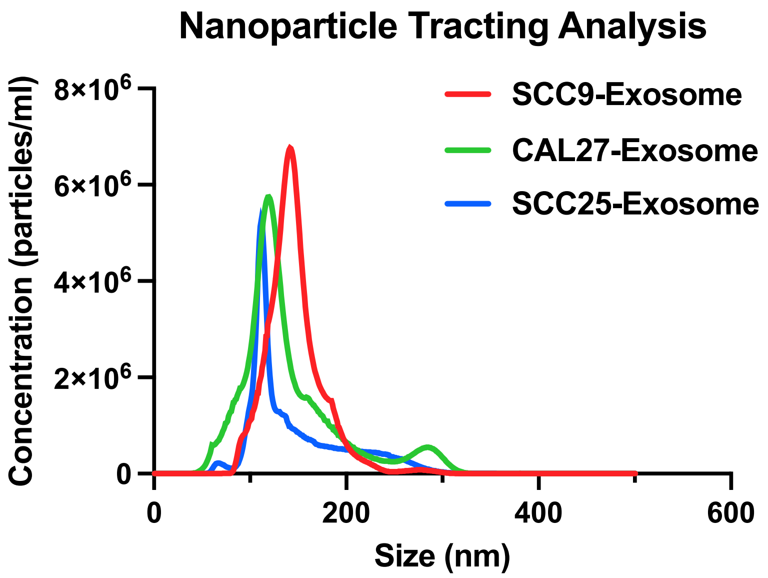

Figure S1 The size and quantity of exosomes were measured using NTA.

Figure S2

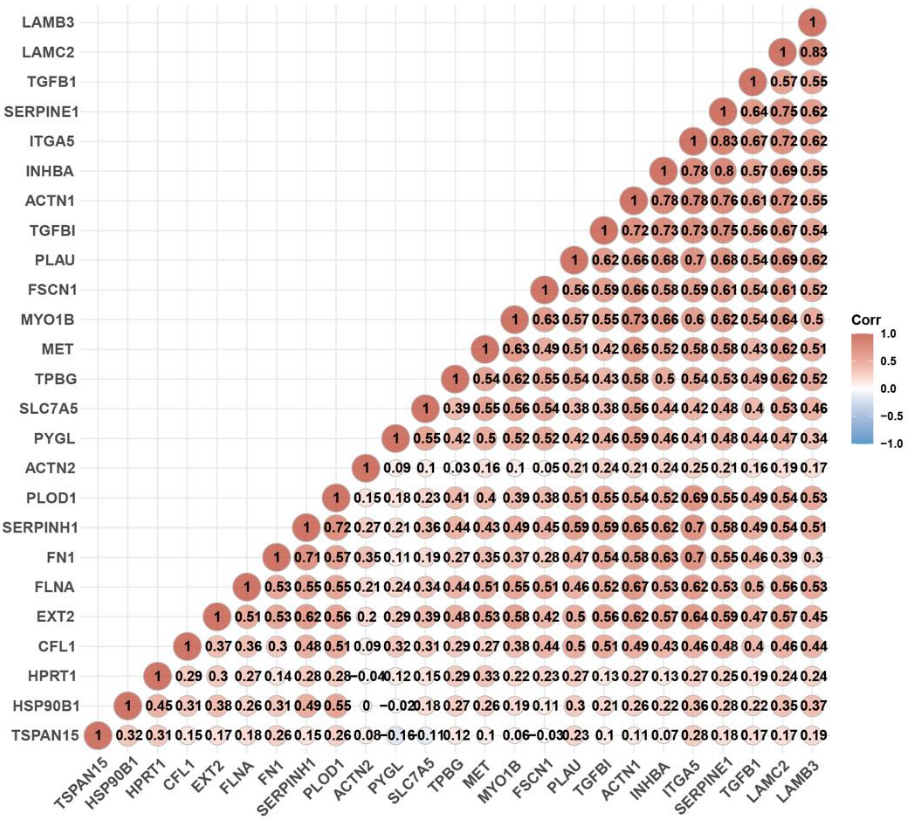

Figure S2 The correlation among 25 prognostic ERGs was assessed via Spearman’s correlation analysis.

Figure S3

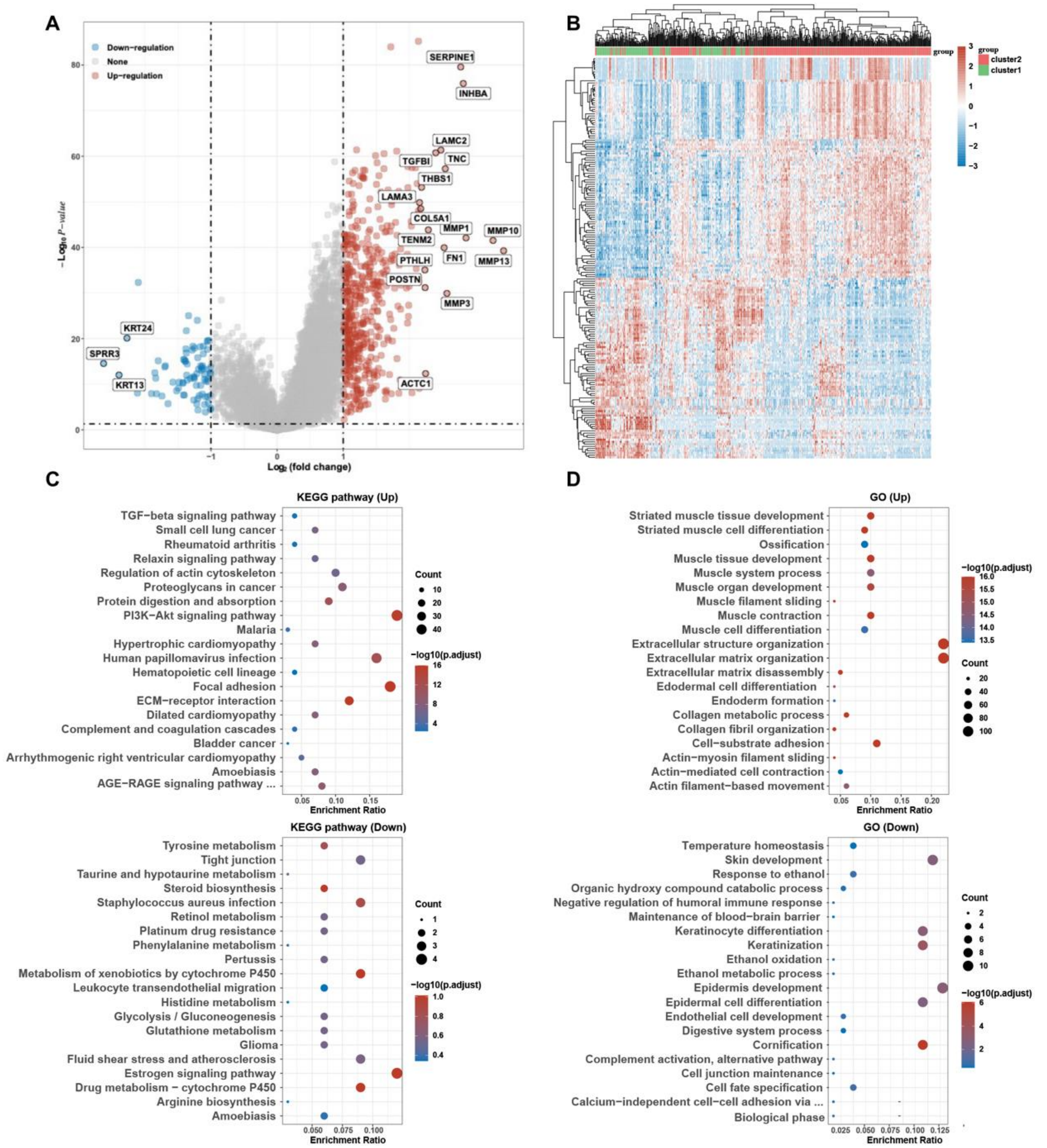

Figure S3 Differential expression genes and functional enrichment analysis of cluster1 and cluster2. (A) The volcano plot was constructed using the fold change＞2 and P-adjust＜0.05. (B) The top 50 up-regulated genes and top 50 down-regulated genes were showed in the heatmap. (C) Bubble graph for KEGG pathway enrichment. (D) Bubble graph for GO enrichment.

Figure S4

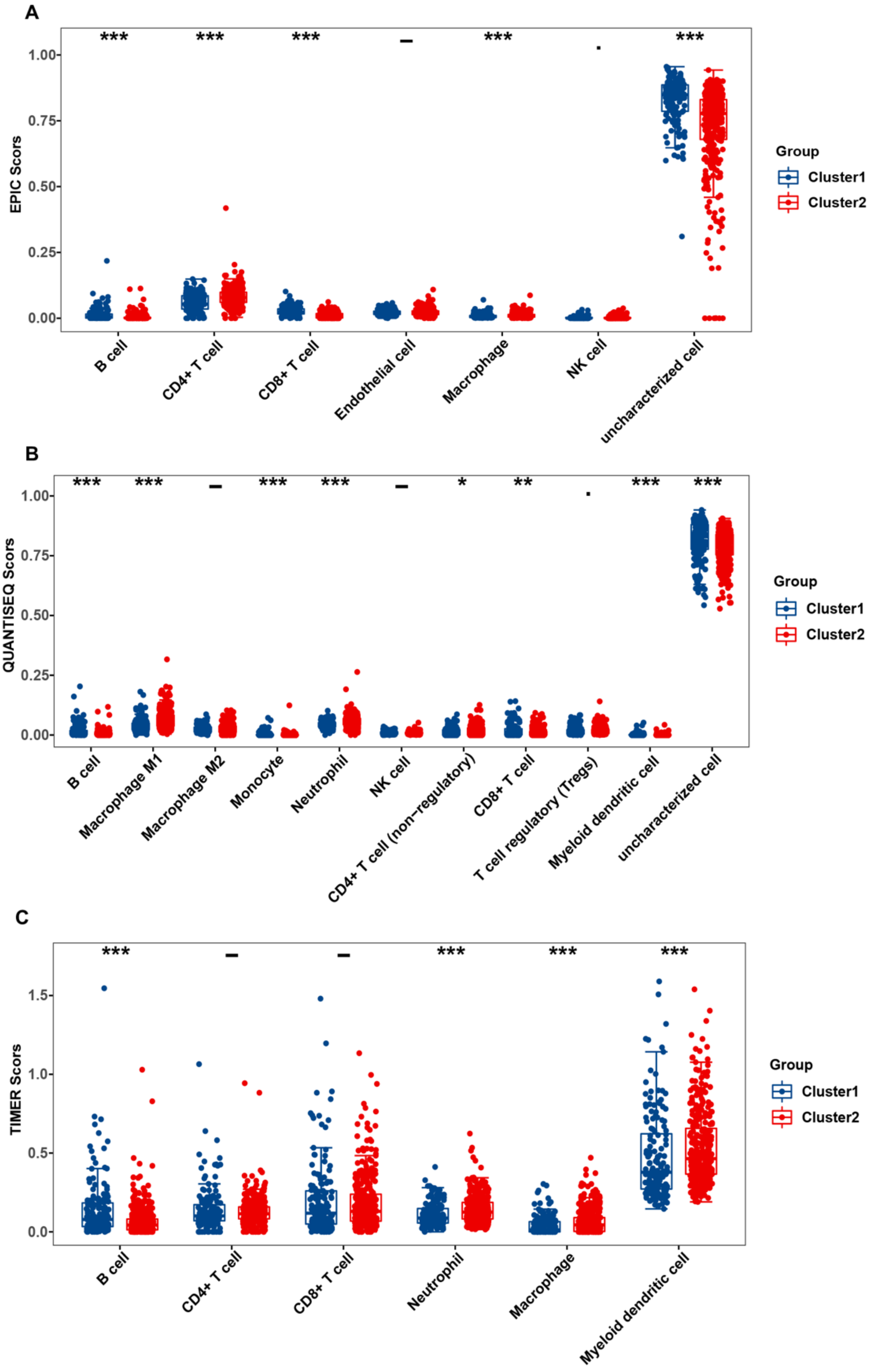

Figure S4 Immune infiltration score of cluster 1 and cluster2 analyzed via four algorithms, EPIC (A), QUANTISEQ (B), TIMER (C).

Figure S5

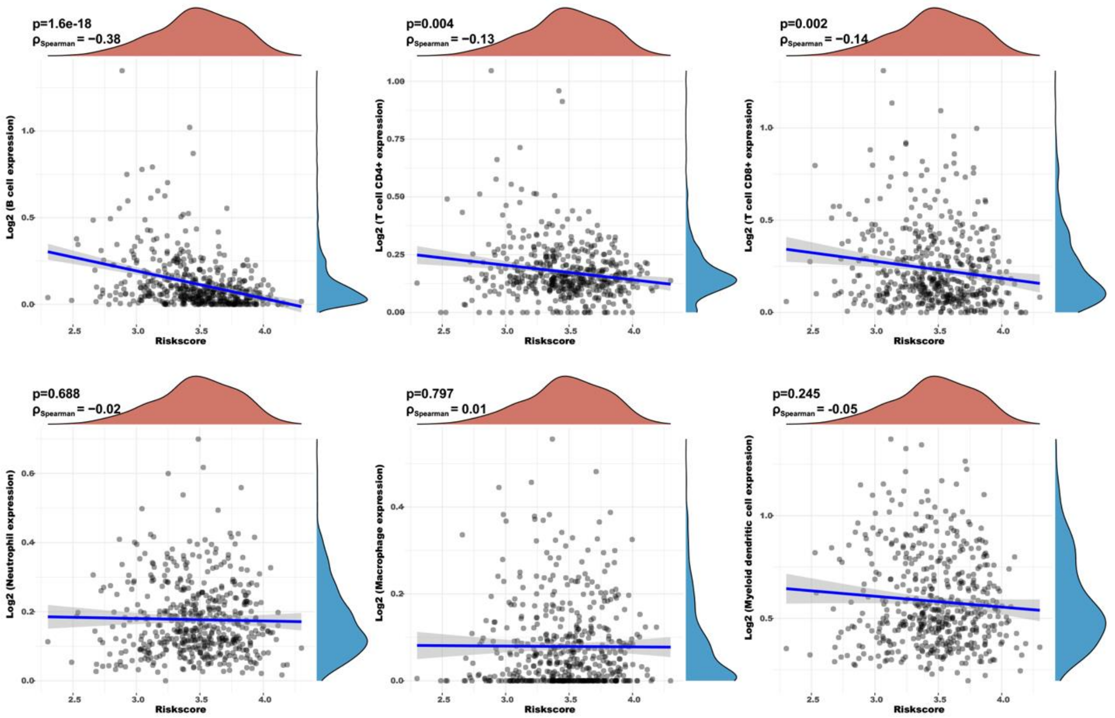

Figure S5 The relationship between the risk model and immune cell infiltration in HNSCC via TIMER algorithm.

Figure S6

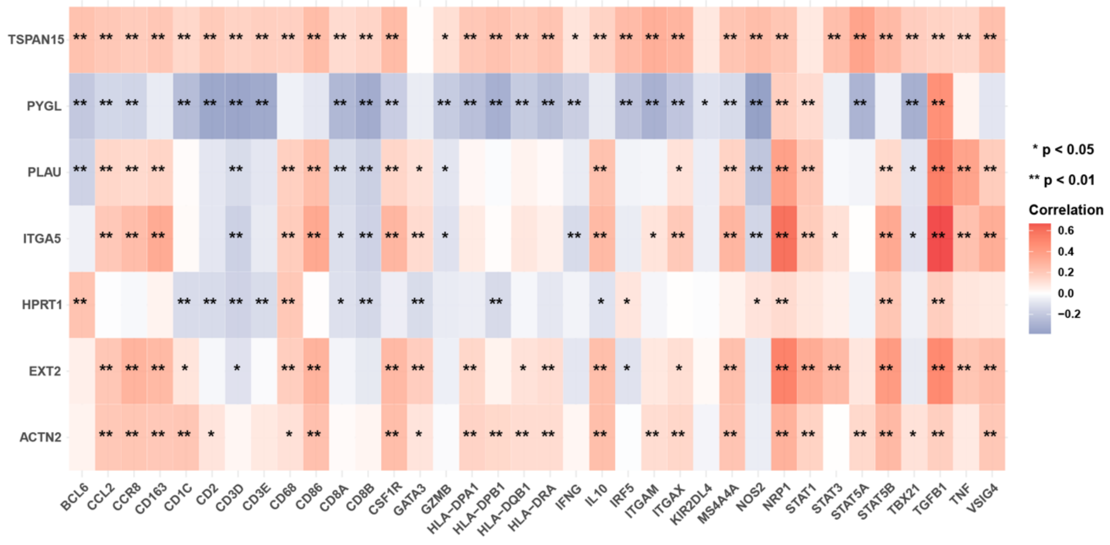

Figure S6 The association between the 7 risky genes and immune cell markers in HNSCC TME.

Figure S7

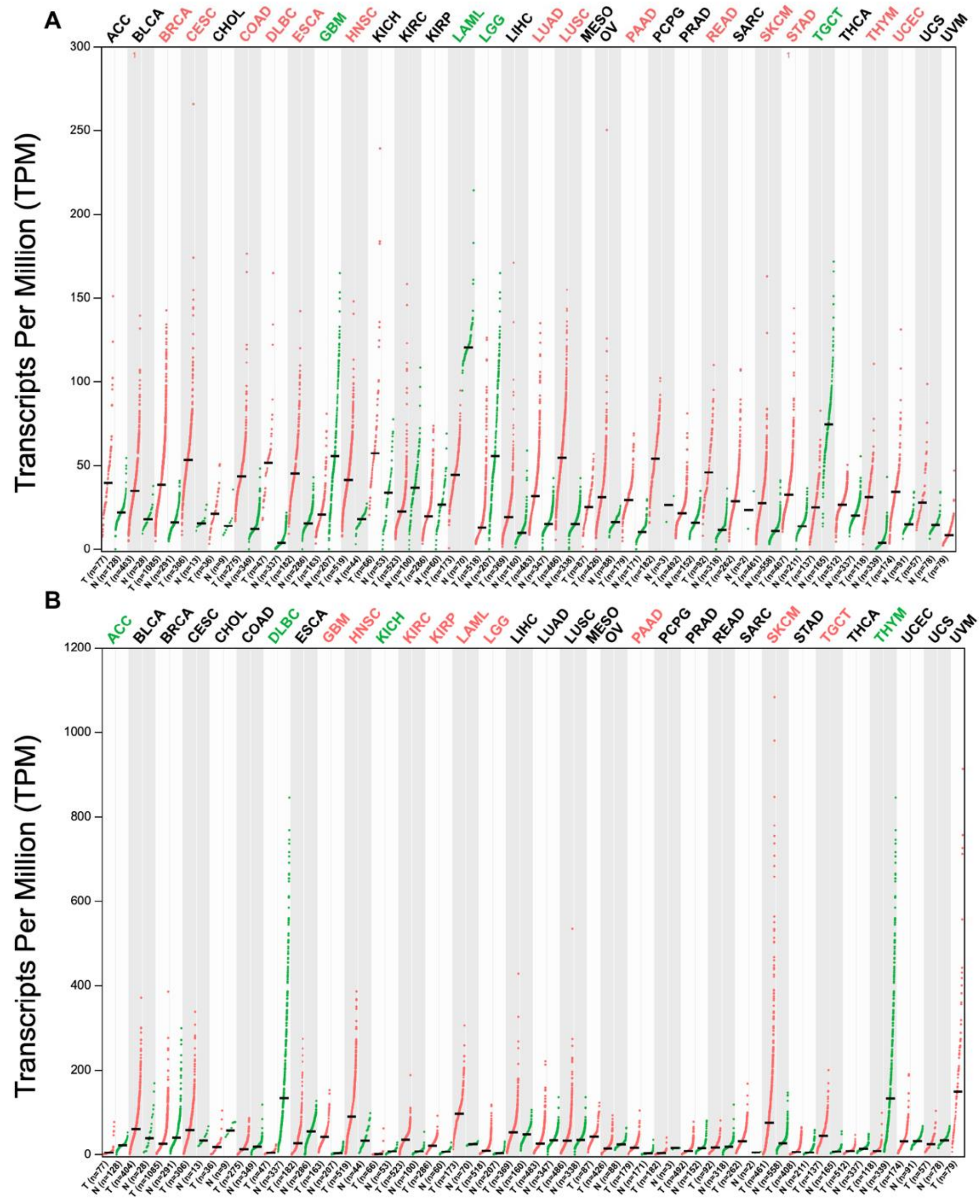

Figure S7 Gene expression level of HPRT1 and PYGL in 33 kinds cancer. (A) HPRT1. (B)PYGL. Red: high expression, Green: low expression.

Figure S8

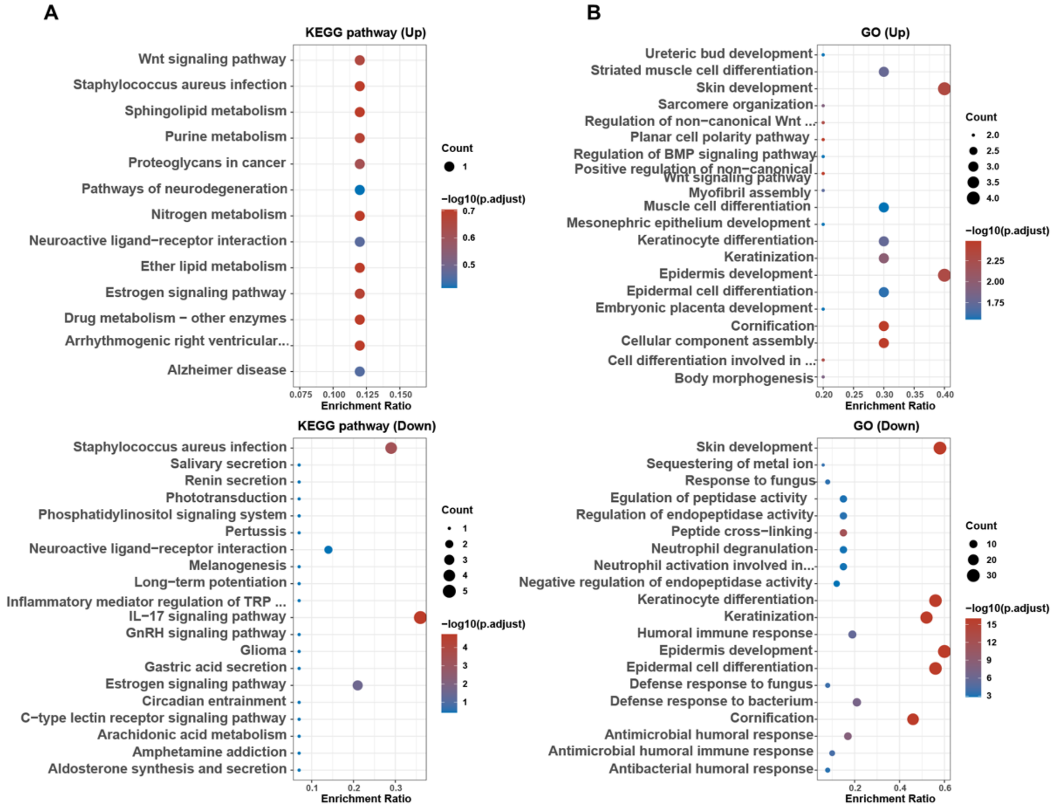

Figure S8 Functional enrichment analysis between HPRT1 high expression group and HPRT1 low expression group in HNSCC. (A)KEGG pathway enrichment analysis. (B) GO functional enrichment analysis.

Figure S9

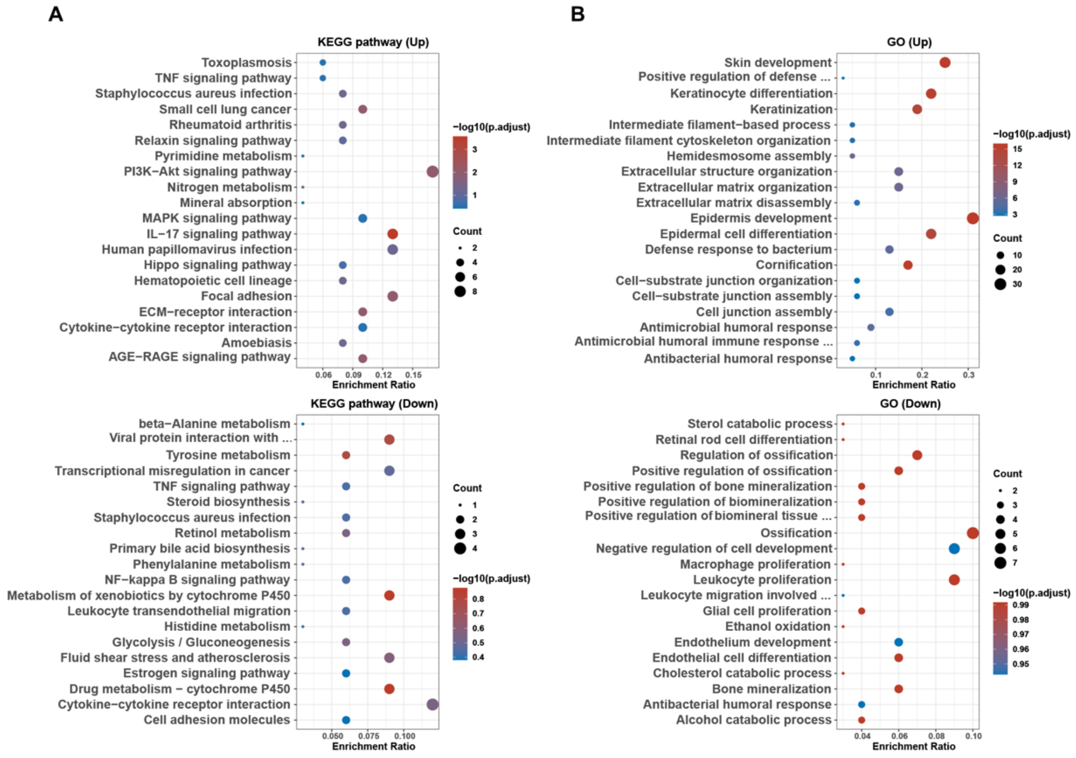

Figure S9 Functional enrichment analysis between PYGL high expression group and PYGL low expression group in HNSCC. (A)KEGG pathway enrichment analysis. (B) GO functional enrichment analysis.

Figure S10

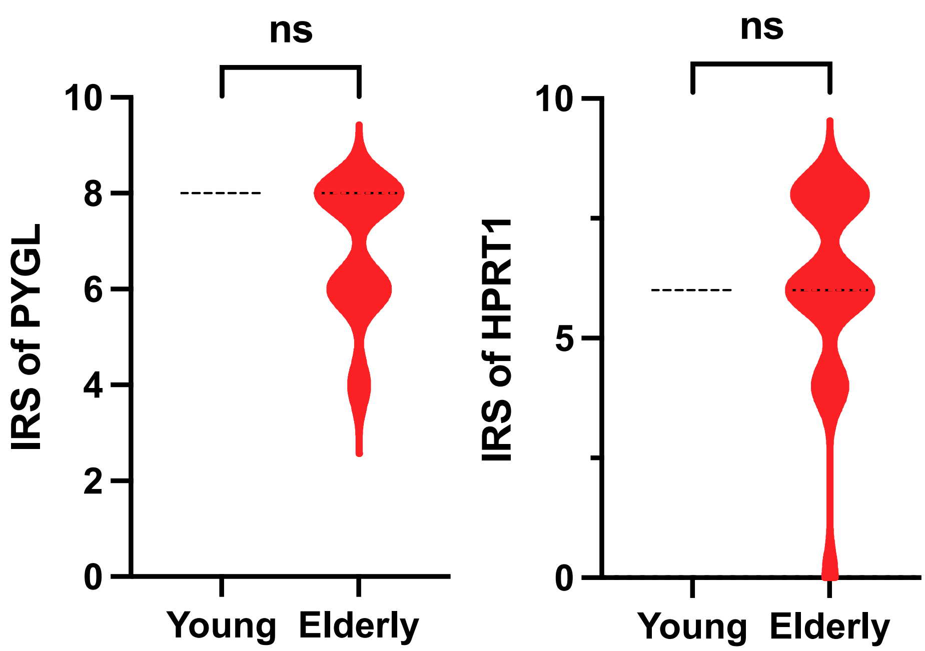

Figure S10 The correlation between HPRT1, PYGL expression and age.

Table S1 The DEGs in TCGA-HNSCC cohort.

DEGs	
GPRIN1	Up
TGFB1	Up
CDCA5	Up
MYBL2	Up
COLGALT1	Up
KIF2C	Up
PKMYT1	Up
BIRC5	Up
UBE2C	Up
CENPA	Up
PLK1	Up

CDC20	Up
MFAP2	Up
CD276	Up
PARP12	Up
TPX2	Up
CFAP251	Up
TK1	Up
COL4A1	Up
SH2D2A	Up
FSCN1	Up
BMP1	Up
CEP55	Up
MRGBP	Up
COL4A2	Up
NETO2	Up
AURKA	Up
HOMER3	Up
CDC25B	Up
COL7A1	Up
MMP9	Up
CDC45	Up
BLOC1S3	Up
CDCA4	Up
CCNB2	Up
SERPINH1	Up
SLC52A2	Up
KPNA2	Up
AUNIP	Up
AURKB	Up
CDC6	Up
C1QTNF6	Up
SHCBP1	Up
ACOT7	Up
UBE2S	Up
KIF4A	Up
PSMB2	Up
RAD51	Up
WDR53	Up
MMP1	Up
LOXL2	Up
CDCA3	Up
USB1	Up

TPBG	Up
PTK7	Up
PRR11	Up
SLC26A6	Up
DLGAP5	Up
PLAU	Up
NRM	Up
LAMC2	Up
HJURP	Up
CKS1B	Up
TMEM132A	Up
TTYH3	Up
TROAP	Up
MMP12	Up
CDKN3	Up
C16orf74	Up
HSD17B6	Up
ARPC1B	Up
MAP4K2	Up
SPAG5	Up
FOXM1	Up
WDR54	Up
MTFR2	Up
MTHFD1L	Up
ZNF707	Up
TACC3	Up
SNX10	Up
TEDC2	Up
PLOD1	Up
KIF18B	Up
EXO1	Up
ISG15	Up
CDCA8	Up
LPCAT1	Up
ORC6	Up
FZD2	Up
TAGLN2	Up
BCL2L12	Up
MELK	Up
NCAPG	Up
AATF	Up
TRIP13	Up

P3H1	Up
NCLN	Up
ARFGAP1	Up
VAV2	Up
FEN1	Up
CDH24	Up
CTHRC1	Up
GTSE1	Up
GNA12	Up
AP5Z1	Up
KIF23	Up
CBX3	Up
CCM2	Up
MMP11	Up
BUB1	Up
RPN1	Up
COL1A1	Up
MSANTD3	Up
PLOD3	Up
CFL1	Up
CA9	Up
TONSL	Up
EXT1	Up
OCIAD2	Up
AGRN	Up
FBXL6	Up
NDC80	Up
EME1	Up
TMEM184B	Up
CCNE1	Up
DTL	Up
ALG3	Up
SNAI2	Up
EFNB1	Up
KIFC1	Up
COL27A1	Up
CDK1	Up
ARTN	Up
NEK2	Up
NCAPH	Up
GINS1	Up
CCNB1	Up

HOXD11	Up
GNB5	Up
RAD54L	Up
AIM2	Up
COL5A1	Up
PRIM2	Up
RFC4	Up
COL5A2	Up
DNMT3B	Up
CIP2A	Up
COL13A1	Up
TGFBI	Up
SLC38A7	Up
NUTF2	Up
CENPO	Up
INHBA	Up
SPINDOC	Up
STX1A	Up
PCLAF	Up
DEPDC1B	Up
ARHGEF1	Up
CCNF	Up
CKS2	Up
CDT1	Up
KIF14	Up
NDE1	Up
IFI6	Up
LY6K	Up
TUBB	Up
SPC24	Up
E2F1	Up
CHST11	Up
RTKN	Up
CYRIB	Up
RUVBL1	Up
IKBIP	Up
CHEK1	Up
ULBP2	Up
ADA	Up
RCC2	Up
ADAMTS2	Up
TXNDC12	Up

CCNA2	Up
IL11	Up
RANBP1	Up
MMP14	Up
HOXD9	Up
CENPN	Up
PSMD2	Up
PFN1	Up
CHTF18	Up
ACOT9	Up
PPP1R18	Up
STC2	Up
C11orf24	Up
SPC25	Up
P3H4	Up
HOXD10	Up
CGAS	Up
UHRF1	Up
MOGS	Up
SLC20A1	Up
ATP6V1C1	Up
TMSB10	Up
CENPI	Up
COL12A1	Up
XRCC3	Up
PLXNA1	Up
BST2	Up
RNASEH2A	Up
KNSTRN	Up
BAX	Up
WDHD1	Up
SAMD1	Up
KIAA0930	Up
IFI27	Up
HMGB3	Up
KIF22	Up
GINS2	Up
ITGA5	Up
SOCS1	Up
MCM2	Up
AGTRAP	Up
CERCAM	Up

FAAP24	Up
ORC1	Up
MED30	Up
FANCI	Up
DDX39A	Up
SPRY4	Up
HAUS8	Up
RACGAP1	Up
NUF2	Up
CDC25C	Up
EIF5A2	Up
TYMP	Up
SLC16A1	Up
SKA1	Up
DCBLD1	Up
LIMK1	Up
HSP90B1	Up
CKAP2L	Up
ASF1B	Up
HOXC13	Up
CHST2	Up
PLAUR	Up
SLC2A1	Up
QSOX2	Up
RGS19	Up
GMNN	Up
HPRT1	Up
SERPINE1	Up
TMEM138	Up
YEATS2	Up
SLC2A6	Up
MARVELD1	Up
UBE2T	Up
ADAM12	Up
MYO1B	Up
KIF18A	Up
PDIA5	Up
IRF3	Up
DSCC1	Up
AP2M1	Up
DNAJB11	Up
PTTG1	Up

SNRPB	Up
PSRC1	Up
RAB34	Up
BRMS1	Up
ACD	Up
MCM10	Up
BUB1B	Up
ATAD2	Up
LMNB2	Up
TRAM2	Up
ENTR1	Up
PRELID3A	Up
PMEPA1	Up
PDIA4	Up
STK3	Up
BRAT1	Up
MARCKSL1	Up
LRFN4	Up
POGLUT2	Up
RAB32	Up
MIIP	Up
IGF2BP2	Up
RRM2	Up
MED15	Up
MCM5	Up
GJC1	Up
COL4A5	Up
TCF3	Up
MORC2	Up
BOP1	Up
PRC1	Up
PTHLH	Up
HOXC9	Up
MTBP	Up
PEDS1	Up
ZWINT	Up
ECE2	Up
SKA2	Up
POLD1	Up
PACC1	Up
TEDC1	Up
TTK	Up

RECQL4	Up
YDJC	Up
IMPDH1	Up
CCN4	Up
DEPDC1	Up
TREM2	Up
MED10	Up
ACP5	Up
NUDT1	Up
RAC2	Up
P4HA1	Up
KLF7	Up
IQGAP3	Up
ANLN	Up
ECT2	Up
FBXO45	Up
MFSD10	Up
XRCC2	Up
MAD2L1	Up
ZIC2	Up
TNFAIP8L1	Up
INF2	Up
FUCA2	Up
UCN2	Up
PTGFRN	Up
RAD51AP1	Up
CENPE	Up
TP53I13	Up
KIF20A	Up
ERF	Up
PFDN2	Up
SLC3A2	Up
EIF2AK2	Up
FST	Up
RTN4R	Up
TIMELESS	Up
SPATS2	Up
GSDME	Up
MKI67	Up
ELF4	Up
SMS	Up
ADAM8	Up

SEMA4F	Up
S1PR5	Up
DTYMK	Up
POLA2	Up
SKA3	Up
CENPF	Up
PSMC3IP	Up
SLC19A1	Up
ABL2	Up
SIAH1	Up
RMI2	Up
CLSPN	Up
DIPK1A	Up
FANCA	Up
ESM1	Up
SLC15A3	Up
E2F7	Up
MFSD12	Up
MCM4	Up
FOXD1	Up
HMMR	Up
KIF3C	Up
HASPIN	Up
CDCA2	Up
STARD4	Up
MOB3A	Up
C1orf216	Up
NCBP2	Up
ENO2	Up
MCM7	Up
PARP14	Up
HOXB7	Up
MAD2L2	Up
NXPH4	Up
ADPGK	Up
PLCG1	Up
HOXD8	Up
COL3A1	Up
CENPL	Up
PIF1	Up
FMNL3	Up
ARHGAP11A	Up

TBC1D31	Up
PGF	Up
TOP2A	Up
RELT	Up
NCS1	Up
FADS3	Up
HTATIP2	Up
TCOF1	Up
ATP1B3	Up
MGME1	Up
CTSV	Up
FANCB	Up
OIP5	Up
IGSF8	Up
XYLT2	Up
KNTC1	Up
PCED1B	Up
RAB31	Up
THY1	Up
PYCR3	Up
CLPTM1L	Up
MND1	Up
PLEK2	Up
LAMB1	Up
TCP11L1	Up
CHPF2	Up
CCT5	Up
NUSAP1	Up
TAP2	Up
DSN1	Up
SFXN3	Up
GPR176	Up
LAMB3	Up
CXCL13	Up
TNFRSF12A	Up
WNT7B	Up
IER5L	Up
PLA2G7	Up
CAMK2N2	Up
HOXA10	Up
CEP131	Up
NEIL3	Up

SPHK1	Up
STAT2	Up
TNFRSF4	Up
RHEBL1	Up
KIF26B	Up
ADAR	Up
PLK4	Up
MAN1B1	Up
HOXA1	Up
GALNT2	Up
PDPN	Up
COL16A1	Up
PIMREG	Up
ADAMTSL2	Up
KRT17	Up
ZFP64	Up
COTL1	Up
SAC3D1	Up
HOXC11	Up
RIPK2	Up
LGALS1	Up
CLIC4	Up
KIF11	Up
SENP5	Up
B3GAT3	Up
SPARC	Up
RCN3	Up
SHFL	Up
IFI35	Up
LRR1	Up
DBF4B	Up
SOX12	Up
CMSS1	Up
STK10	Up
FGD6	Up
NUDCD1	Up
CHN1	Up
ERCC6L	Up
PIK3CD	Up
CKAP2	Up
HROB	Up
COL1A2	Up

B3GNT4	Up
PVR	Up
NMI	Up
FXYD5	Up
FOXF2	Up
GRIN2D	Up
COL10A1	Up
EMC1	Up
CTSC	Up
CYP27B1	Up
PDLIM7	Up
COL6A1	Up
GALNT18	Up
CCSAP	Up
SCRN1	Up
BICD1	Up
LAMA3	Up
ITGA6	Up
MSN	Up
ASPM	Up
DPF1	Up
LPAR2	Up
STK17A	Up
MYO10	Up
FNDC3B	Up
GALNS	Up
RHBDD3	Up
RHOC	Up
JOSD1	Up
VMP1	Up
MTERF3	Up
RPA3	Up
H4C9	Up
COL5A3	Up
RPS6KA4	Up
FKBP9	Up
MTFR1	Up
COL6A3	Up
ODF2	Up
POC1A	Up
CAVIN3	Up
NAGS	Up

SHOX2	Up
NEMP1	Up
ZNF114	Up
HOXC6	Up
POLE2	Up
OASL	Up
FAM167B	Up
C1orf112	Up
C18orf54	Up
ADCK5	Up
APLN	Up
SLC39A4	Up
POLR2H	Up
FANCE	Up
MMD	Up
P4HA2	Up
NRIP3	Up
PCNA	Up
STMN1	Up
LASP1	Up
DCLRE1B	Up
H3C10	Up
IGFBP7	Up
LRRC8C	Up
NREP	Up
CENPM	Up
ACTL6A	Up
VRK1	Up
FSTL3	Up
MCUB	Up
DDX11	Up
UAP1L1	Up
PPT1	Up
ARHGEF39	Up
POSTN	Up
SKP2	Up
STIL	Up
MRPL47	Up
C20orf27	Up
ANGPT2	Up
CDK2	Up
EVA1B	Up

ZNF281	Up
GNGT1	Up
GLA	Up
ADAMTS12	Up
BAK1	Up
GGH	Up
MASTL	Up
CORO1C	Up
POLQ	Up
SLC44A1	Up
SPOCD1	Up
MINPP1	Up
NAT14	Up
PHLDB2	Up
FADS1	Up
CDH3	Up
KAT2A	Up
IFI44	Up
TOP1MT	Up
APP	Up
DUSP14	Up
FOXS1	Up
PCSK9	Up
PROCR	Up
ZWILCH	Up
FAP	Up
FAM111A	Up
ADAMTS7	Up
RSRC1	Up
TMEM106C	Up
TAF1A	Up
TAPBP	Up
EPSTI1	Up
PXN	Up
KHDC1	Up
LGALS3BP	Up
XPR1	Up
MMP3	Up
LSR	Up
MCM6	Up
KNL1	Up
MMP13	Up

TEAD2	Up
MARK1	Up
RFWD3	Up
MIF	Up
CSGALNACT2	Up
SLC11A1	Up
DDX60L	Up
RBP1	Up
PTK2	Up
NCF2	Up
TYMS	Up
DIAPH3	Up
TM4SF19	Up
DNA2	Up
CDK4	Up
TTPAL	Up
PLBD2	Up
ASAP1	Up
STX2	Up
NID1	Up
SQLE	Up
LAPTM4B	Up
GMPS	Up
HOXC4	Up
C4orf48	Up
NASP	Up
PIEZO1	Up
APBA2	Up
FANCG	Up
SLC25A22	Up
COL6A2	Up
BRCA1	Up
NCAPD2	Up
PSMG3	Up
EPHB4	Up
EPHB2	Up
MFHAS1	Up
ITGB4	Up
C6orf141	Up
TCIRG1	Up
DBF4	Up
SGO2	Up

TMC7	Up
H2AC11	Up
ARID3A	Up
TAP1	Up
SP100	Up
SHC1	Up
L3HYPDH	Up
KLHL5	Up
CEP72	Up
OSBPL3	Up
CCDC14	Up
CHPF	Up
FN1	Up
H2AZ1	Up
MICB	Up
TUBB3	Up
PTDSS1	Up
LY6E	Up
H1-10	Up
DONSON	Up
CALU	Up
FJX1	Up
H2BC5	Up
FAM219A	Up
GSDMD	Up
SMTN	Up
ADAMTS14	Up
PXDN	Up
DNMT1	Up
PDE7A	Up
ICAM5	Up
SNAPC1	Up
IFI16	Up
ITGA3	Up
LUM	Up
PAQR4	Up
NLRC5	Up
H2BC12	Up
HMGA2	Up
HTRA3	Up
IRF9	Up
GANAB	Up

MICAL2	Up
MTHFD2	Up
MB21D2	Up
CIBAR1	Up
MIS18A	Up
LIMD2	Up
POFUT1	Up
DTX3L	Up
CHST15	Up
BEX3	Up
FBLIM1	Up
CBLB	Up
DUSP9	Up
EXOSC4	Up
CCDC77	Up
STON2	Up
TRAIP	Up
SUGCT	Up
ENAH	Up
GREM1	Up
OAS2	Up
ITGA1	Up
BID	Up
TMEM44	Up
APOL1	Up
SLC36A1	Up
LAMC1	Up
ABCD1	Up
GPR153	Up
GPX8	Up
TNFRSF10B	Up
ANP32E	Up
TBL1XR1	Up
CENPW	Up
BLM	Up
BORA	Up
NELL2	Up
FEZ1	Up
NCAPG2	Up
ATP2C1	Up
BNC1	Up
PLPP4	Up

HLA-A	Up
OAS3	Up
HERC5	Up
SH2D5	Up
SLC1A5	Up
FERMT1	Up
H2BC9	Up
ZIC5	Up
FLNA	Up
PRNP	Up
FANCC	Up
CNPY4	Up
CHAF1A	Up
FKBP10	Up
CCL11	Up
APOBEC3B	Up
CHEK2	Up
ASPHD2	Up
PBK	Up
LAMP5	Up
EN1	Up
TPST1	Up
C12orf75	Up
GAST	Up
RFC5	Up
SCG5	Up
RAD21	Up
BCAS4	Up
PHF19	Up
ANXA5	Up
FAM89A	Up
USP18	Up
HOXC8	Up
HTRA1	Up
FMNL2	Up
HOXC10	Up
APMAP	Up
PTPN12	Up
ZNF697	Up
HELZ2	Up
RFLNB	Up
UBE2L6	Up

PAK2	Up
H2AX	Up
LRRC8D	Up
TREM1	Up
DNAH17	Up
SGO1	Up
CPNE1	Up
DDIAS	Up
SPP1	Up
LOXL3	Up
CNPY3	Up
SEMA7A	Up
STAT1	Up
CENPH	Up
FBXO5	Up
CPXM1	Up
CTSZ	Up
TRIM59	Up
H2AC13	Up
C2	Up
TSPAN9	Up
CLSTN1	Up
RSAD2	Up
HLA-B	Up
DNASE2	Up
TSPAN10	Up
FADD	Up
TOX2	Up
THEMIS2	Up
PSMB9	Up
CAD	Up
KREMEN2	Up
NUP107	Up
CTLA4	Up
TNFRSF25	Up
GPR4	Up
CBX2	Up
BGN	Up
PML	Up
PNMA1	Up
XXYLT1	Up
JAG1	Up

MCM3	Up
SLC16A3	Up
FZD6	Up
PLXND1	Up
MPP3	Up
PDGFRB	Up
EMP3	Up
DCBLD2	Up
UBXN7	Up
GALNT10	Up
FKBP14	Up
B3GNT9	Up
HLA-F	Up
TCF19	Up
SKIL	Up
AMIGO2	Up
EHD2	Up
NOX4	Up
MET	Up
AJUBA	Up
CHST7	Up
ATAD5	Up
VOPP1	Up
PLD6	Up
LAMA5	Up
HLA-C	Up
EXT2	Up
NRSN2	Up
FAM83H	Up
TMC8	Up
AEBP1	Up
CLEC11A	Up
LTBP1	Up
MCM8	Up
CD109	Up
DLX1	Up
PDGFA	Up
PLAC1	Up
MSC	Up
FBXO41	Up
GLS	Up
CDR2	Up

JAG2	Up
HEYL	Up
MSH6	Up
HELLS	Up
LRRC3	Up
SLC39A13	Up
SIGMAR1	Up
THSD1	Up
BRCA2	Up
CYP27C1	Up
MMP17	Up
NFKBIE	Up
APBB2	Up
HOXD13	Up
C3orf52	Up
DVL3	Up
GALNT6	Up
TMEM200B	Up
OSR2	Up
LRP8	Up
GNAI1	Up
TRPM2	Up
LHX5	Up
CDC7	Up
ERFE	Up
FTL	Up
H2BC17	Up
TRIB3	Up
FOXP3	Up
NAV1	Up
PTPRK	Up
CMTM3	Up
TICRR	Up
TMTC3	Up
B4GALNT1	Up
IL24	Up
MMP19	Up
RELB	Up
SLC7A5	Up
RGS4	Up
CCDC24	Up
ITM2C	Up

RRAS2	Up
PRIM1	Up
KDELR3	Up
OLFML2B	Up
LHFPL2	Up
RFC3	Up
TNFRSF9	Up
ESCO2	Up
TMEM92	Up
PLOD2	Up
NID2	Up
CDK18	Up
GBP5	Up
TMEM263	Up
P3H2	Up
BMP8A	Up
ITPR3	Up
RTTN	Up
CASK	Up
HES4	Up
HENMT1	Up
C1QL1	Up
GPR39	Up
DDX58	Up
CIT	Up
KIRREL1	Up
GINS4	Up
IL4I1	Up
PGGHG	Up
KLHDC7B	Up
NLGN2	Up
CSF2	Up
AFAP1L2	Up
F2RL2	Up
TMEM158	Up
CTSL	Up
LIG1	Up
DDX60	Up
LRP12	Up
TOPBP1	Up
TNFSF4	Up
TNFRSF18	Up

IRF7	Up
CENPU	Up
NRBP2	Up
COL4A6	Up
BRIP1	Up
LAMP3	Up
PCDH17	Up
KHDC1L	Up
IL15RA	Up
NPNT	Up
GNLY	Up
WNT2	Up
PSTPIP1	Up
WDR76	Up
ITGAX	Up
MMP10	Up
GFI1	Up
CENPK	Up
SLC1A4	Up
ATP13A3	Up
IGSF3	Up
SLC17A9	Up
FCGR2A	Up
F2R	Up
MDFI	Up
PFKFB4	Up
CLEC7A	Up
GPR161	Up
BFSP1	Up
TENM2	Up
P4HA3	Up
ID3	Up
LHB	Up
HTR7	Up
KIFC2	Up
CELSR3	Up
ZNF469	Up
NSD2	Up
SMC2	Up
COL11A1	Up
HPS3	Up
NKD2	Up

SCD5	Up
TFRC	Up
GGT5	Up
RDH16	Up
NMB	Up
KIF20B	Up
IL2RA	Up
DEPDC7	Up
ACVR1	Up
ASPN	Up
CAV1	Up
CDR2L	Up
FCGR3A	Up
RAI14	Up
RFTN1	Up
IFIT3	Up
SLAMF8	Up
SMOX	Up
CLCN2	Up
TNC	Up
NTM	Up
HOXA11	Up
XCL1	Up
LRRC15	Up
OLFML2A	Up
TRIO	Up
H2AC8	Up
CDCA7L	Up
PAG1	Up
CDH11	Up
ENTPD7	Up
IBSP	Up
FAM111B	Up
DPY19L1	Up
LBH	Up
NDRG1	Up
TUBA1B	Up
CCNP	Up
SEC61G	Up
RUNX3	Up
XAF1	Up
HAP1	Up

BLACAT1	Up
WARS1	Up
APOC1	Up
TP63	Up
EZH2	Up
VEGFA	Up
PRR5L	Up
BASP1	Up
ERVMER34-1	Up
ABCG1	Up
TDO2	Up
SLC52A1	Up
PDCD1LG2	Up
GPR158	Up
PPP1R14C	Up
APOL2	Up
SCARB1	Up
IFITM3	Up
MYO5A	Up
EMILIN1	Up
NUP62CL	Up
PILRA	Up
ZP3	Up
PROC	Up
SPAG4	Up
SMIM3	Up
EIF4EBP1	Up
ROR2	Up
IL7R	Up
PPP4R4	Up
KCNJ8	Up
TGFB1I1	Up
SYDE1	Up
TFAP2E	Up
FOXL1	Up
ST6GALNAC2	Up
HAPLN3	Up
IFIH1	Up
FBN2	Up
H2BC11	Up
GJB3	Up
TENM3	Up

DLX5	Up
NRG1	Up
COPZ2	Up
EMILIN2	Up
TBX2	Up
PLSCR1	Up
NEK6	Up
CNGB1	Up
IFIT2	Up
ARSI	Up
THBS2	Up
DDIT3	Up
GPR68	Up
LMNB1	Up
BAIAP2L2	Up
CMPK2	Up
IFI44L	Up
SGPP1	Up
CAV2	Up
DLX6	Up
SLC39A14	Up
LAMA4	Up
SULF1	Up
KIF15	Up
CD70	Up
GPC1	Up
PRKDC	Up
DSG2	Up
ADTRP	Up
MCAM	Up
TSPAN15	Up
CEACAM19	Up
MATN3	Up
ECE1	Up
OSCAR	Up
ANO1	Up
LARGE2	Up
RASL11B	Up
RTP4	Up
ADAM19	Up
COL18A1	Up
CHSY3	Up

EDDM13	Up
TINAGL1	Up
HMGB2	Up
SMC4	Up
BMF	Up
CALD1	Up
HES2	Up
ICOS	Up
ACTN1	Up
RGS20	Up
SDS	Up
CD300LF	Up
CXCL10	Up
PABPC1L	Up
PLEKHG4B	Up
FADS2	Up
GLIPR1	Up
MT1F	Up
APOE	Up
SLC7A8	Up
GPSM1	Up
GJB4	Up
CDK5R1	Up
HAGHL	Up
TRPV3	Up
MYO1G	Up
ETS1	Up
DRAM1	Up
FCHO1	Up
CDK6	Up
LYPD1	Up
IGFBP3	Up
LIMA1	Up
HLTF	Up
EN2	Up
LAG3	Up
COL8A1	Up
ITGAV	Up
MMP2	Up
SLC4A3	Up
HK3	Up
IGF2BP3	Up

MEIS3	Up
IFIT1	Up
FGFR4	Up
CSPG4	Up
SCARF2	Up
HIP1	Up
HIF1A	Up
TIGIT	Up
IFITM1	Up
RNASE10	Up
TMEM204	Up
DDIT4	Up
GLIS1	Up
LOX	Up
GJA1	Up
GAREM2	Up
MELTF	Up
NFE2L3	Up
CD86	Up
FCER1G	Up
PAEP	Up
ARSJ	Up
ANKRD13B	Up
PDGFB	Up
FAM241B	Up
PYGL	Up
TNFAIP3	Up
SH2B3	Up
GLIS2	Up
DLX2	Up
SHISAL1	Up
HOXB9	Up
EVA1A	Up
ICAM1	Up
ASNS	Up
CREG2	Up
FOXL2	Up
IQCG	Up
ZNF367	Up
EGFL6	Up
LTO1	Up
IL1A	Up

PAPLN	Up
RENBP	Up
MISP	Up
BAG2	Up
TNFAIP6	Up
RASGEF1A	Up
TRAF1	Up
SLAMF9	Up
CXCL9	Up
CXCL11	Up
RPL39L	Up
GOLIM4	Up
ITGA11	Up
DNM1	Up
CDCA7	Up
SLC7A7	Up
MEST	Up
CCL5	Up
MYH10	Up
STRA6	Up
STEAP1B	Up
ZBP1	Up
MX2	Up
NRP2	Up
TWIST1	Up
CSRP2	Up
TGFB3	Up
FNDC1	Up
CILP2	Up
SLC12A8	Up
TMEM97	Up
CELSR1	Up
IL18BP	Up
VCAN	Up
KCNE4	Up
WNT7A	Up
TNFSF9	Up
SDK2	Up
FOLR3	Up
TSHZ3	Up
GNG8	Up
SLC28A3	Up

GUCY1B1	Up
C1S	Up
HLA-G	Up
NEFL	Up
ODC1	Up
GSDMB	Up
CPNE7	Up
IL12RB2	Up
SULF2	Up
CTSK	Up
GPNMB	Up
PRTFDC1	Up
ITPKA	Up
COL22A1	Up
SEL1L3	Up
JAK3	Up
IGSF9	Up
RCOR2	Up
ABCC1	Up
GABRE	Up
PRAME	Up
BCAT1	Up
CCL3	Up
LAYN	Up
CXCL8	Up
SIGLEC12	Up
CSAG3	Up
HAVCR2	Up
PPFIA1	Up
ADAMDEC1	Up
HAS3	Up
SLCO1B3	Up
CDK14	Up
CALHM6	Up
PCDHGC5	Up
PNCK	Up
ADM	Up
OLR1	Up
KRT9	Up
SLFN11	Up
LTBP2	Up
IGFL2	Up

BCL2A1	Up
LILRB4	Up
LHX2	Up
OAS1	Up
FLRT2	Up
H4C8	Up
ARHGAP4	Up
FOXRED2	Up
FAT1	Up
VEGFC	Up
NUAK1	Up
UPP1	Up
IGF2BP1	Up
IDO1	Up
LFNG	Up
H3C4	Up
GBP1	Up
MST1R	Up
GZMB	Up
CSAG2	Up
IL27RA	Up
C5AR1	Up
SYNGR3	Up
NUDT11	Up
TNFSF10	Up
LAMA1	Up
TNS4	Up
LPAR3	Up
SDK1	Up
IL36G	Up
AQP9	Up
SEMA3C	Up
JADE2	Up
RAC3	Up
FAT2	Up
CD248	Up
MX1	Up
PI15	Up
NT5E	Up
C1QTNF1	Up
TOR4A	Up
FOXC2	Up

ACKR3	Up
CPA6	Up
PKP1	Up
BMP2	Up
SERPINA1	Up
IRX4	Up
ASPHD1	Up
KIAA1549L	Up
IL32	Up
NPW	Up
ASCL2	Up
KYNU	Up
NOTUM	Up
S100A2	Up
RHEX	Up
SCD	Up
DKK1	Up
RHBDL1	Up
RASD2	Up
ABCC5	Up
FPR3	Up
SCNN1D	Up
H2BC8	Up
ETV4	Up
OLFM2	Up
SERPINE2	Up
STC1	Up
PRSS23	Up
GFPT2	Up
MDK	Up
CHST1	Up
SPON2	Up
CRISPLD2	Up
SLC6A2	Up
SFN	Up
KRT16	Up
KRT5	Up
OSM	Up
CD7	Up
BATF2	Up
PADI3	Up
LURAP1L	Up

LTB	Up
PFN2	Up
PIM2	Up
HKDC1	Up
MXRA8	Up
CCL20	Up
RPE65	Up
DKK3	Up
IL1B	Up
CLMP	Up
GPX7	Up
DHCR7	Up
HAS2	Up
SYNDIG1	Up
GDPD2	Up
HORMAD1	Up
MAGEA6	Up
SLC38A5	Up
GOLGA7B	Up
MAGEA3	Up
PRF1	Up
CTXN1	Up
LOXL1	Up
BCAM	Up
SEZ6L2	Up
CARD11	Up
CNTNAP2	Up
MAGEA4	Up
CSAG1	Up
GRP	Up
CDKN2A	Up
LEMD1	Up
SOST	Up
WNT10A	Up
CASP14	Up
GOLGA8A	Up
CCL18	Up
PCOLCE	Up
EGLN3	Up
AMTN	Up
MMP28	Up
COL17A1	Up

ABCA12	Up
EPCAM	Up
FIBCD1	Up
IGFL3	Up
ITGB6	Up
DERL3	Up
NGF	Up
CHIT1	Up
GTSF1	Up
CALB1	Up
HEY1	Up
CCNA1	Up
ADAMTS15	Up
FABP6	Up
FAM83A	Up
PTPRZ1	Up
CLCA2	Up
TMEM45A	Up
KRT6A	Up
ISLR	Up
EDIL3	Up
KRT75	Up
LCE1F	Up
IL20RB	Up
KRT6B	Up
FBXO2	Up
SLC7A11	Up
MAGEA11	Up
C1QB	Up
SPOCK1	Up
FOXE1	Up
BARX1	Up
ADAM23	Up
C1orf68	Up
ASPRV1	Up
UCHL1	Up
WFDC5	Up
OR2I1P	Up
MMP7	Up
MXRA5	Up
SHISA2	Up
KRT14	Up

C1QTNF12	Up
NDUFA4L2	Up
PTGS2	Up
S100A7A	Up
MAGEB2	Up
FABP5	Up
DSC1	Up
PRR9	Up
CXCL1	Up
CST1	Up
SPINK6	Up
LCE3D	Up
SFRP4	Up
SPRR2G	Up
IGLL5	Up
WFDC12	Up
S100A7	Up
KRTDAP	Up
NRG2	Down
MYOC	Down
ADH1B	Down
CRISP3	Down
PLIN1	Down
CIDEC	Down
GPD1	Down
HMGCS2	Down
MYZAP	Down
SLC27A6	Down
ARSF	Down
ATP6V0A4	Down
TMEM132C	Down
FAM107A	Down
CAB39L	Down
AQP7	Down
EDN3	Down
SH3BGRL2	Down
GYS2	Down
TRARG1	Down
CYP2F1	Down
PLIN4	Down
SLC38A3	Down
FMO2	Down

FAM3D	Down
CFTR	Down
C9orf152	Down
RORC	Down
C16orf89	Down
PEBP4	Down
GPD1L	Down
CCL14	Down
GREM2	Down
MUC21	Down
KLHDC7A	Down
PLIN5	Down
PPP1R1A	Down
CYP4B1	Down
CGNL1	Down
NDRG2	Down
CLEC3B	Down
AQP4	Down
MYRIP	Down
ZBTB16	Down
COBL	Down
TMEM238L	Down
ECRG4	Down
STATH	Down
ESRRG	Down
NUCB2	Down
MAPT	Down
ANGPTL1	Down
CRYM	Down
KRT40	Down
GNA14	Down
SORBS2	Down
SLC6A1	Down
SMIM5	Down
GPT	Down
MAL	Down
TMPRSS11B	Down
PIP	Down
DYNAP	Down
TF	Down
FUT6	Down
AGFG2	Down

THRSP	Down
LPO	Down
SLC2A4	Down
GGTA1	Down
CCDC160	Down
MT1A	Down
SMCO1	Down
DHRS7C	Down
CIDEA	Down
VSIG2	Down
RRAGD	Down
TIMP4	Down
ACOX2	Down
PRH2	Down
KAT2B	Down
FNDC5	Down
ETFDH	Down
MYLK3	Down
FAM189A2	Down
CPEB3	Down
SCIN	Down
PPP1R3C	Down
PAIP2B	Down
KRT4	Down
SPX	Down
ABCA8	Down
SELENBP1	Down
EMP1	Down
SLC16A7	Down
CAPN5	Down
FAM3B	Down
TMEM213	Down
ZG16B	Down
SORBS1	Down
PDK4	Down
CRNN	Down
MUC7	Down
ASB11	Down
FBXO40	Down
RNF224	Down
PYGM	Down
PRH1	Down

PLA2G2A	Down
PLAC9	Down
LRRC2	Down
TMEM100	Down
SLC5A8	Down
HPGD	Down
EXTL1	Down
SLC4A4	Down
CHST9	Down
ARHGEF10L	Down
PRKN	Down
DEPTOR	Down
PI16	Down
CDNF	Down
PLP1	Down
LAMB4	Down
SPNS2	Down
NT5C1A	Down
FAM149A	Down
ENDOU	Down
FAM240C	Down
MINDY1	Down
PERM1	Down
MUC22	Down
DLK1	Down
LRRC26	Down
PRB3	Down
FBP2	Down
C6orf58	Down
NR3C2	Down
GPX3	Down
SASH1	Down
KLF15	Down
KRT32	Down
CEACAM1	Down
MGLL	Down
ASB12	Down
SAMD5	Down
SLC13A2	Down
CAPN14	Down
CHRDL1	Down
PPARG	Down

CA3	Down
PEG3	Down
ZNF844	Down
PPARGC1A	Down
ITM2A	Down
UBL3	Down
C7	Down
GPT2	Down
CXCR2	Down
TLE2	Down
FAM166B	Down
CLCNKB	Down
LDHD	Down
SCNN1B	Down
C15orf62	Down
CYP3A5	Down
KRT33A	Down
FABP7	Down
KLHL33	Down
GJB1	Down
CA6	Down
CHRM1	Down
AQP5	Down
PRR4	Down
HSPB6	Down
STRIT1	Down
TCEAL2	Down
GDF10	Down
AOX1	Down
BARX2	Down
CKMT2	Down
CAPN6	Down
ANXA9	Down
CLCA4	Down
TNXB	Down
MPC1	Down
COX7A1	Down
ABRA	Down
ATP13A4	Down
SHROOM3	Down
NFIX	Down
CFD	Down

F10	Down
MUC15	Down
PPP1R3A	Down
PGM5	Down
KRT78	Down
HLF	Down
ALDH1L1	Down
CEACAM7	Down
B3GNT6	Down
HOPX	Down
CHAD	Down
KRT36	Down
PLEKHA6	Down
METTL7A	Down
S100A1	Down
AMOT	Down
FRZB	Down
GMDS	Down
TM7SF2	Down
CTTNBP2	Down
GDPD3	Down
SPATA18	Down
SLC16A6	Down
BCAS1	Down
PAX9	Down
DKK4	Down
SLC6A4	Down
GCNT3	Down
MAML3	Down
MAB21L4	Down
CRTAC1	Down
CASQ1	Down
MKRN2OS	Down
AMPD1	Down
UPK1A	Down
LTF	Down
ANK2	Down
ASB10	Down
SNX31	Down
ANO5	Down
CYP2J2	Down
MMRN1	Down

LRRN4CL	Down
BMP3	Down
STAC2	Down
SCARA5	Down
APOD	Down
FCER1A	Down
MFSD6L	Down
PADI1	Down
EPHX2	Down
MYOM1	Down
AIF1L	Down
TJP3	Down
AGBL1	Down
ACKR1	Down
TGM3	Down
TSPAN8	Down
CAVIN2	Down
VIT	Down
CLU	Down
EPB41L4A	Down
ANKRD35	Down
PCDH1	Down
PRSS27	Down
FYCO1	Down
BOC	Down
CYP4F12	Down
CNTFR	Down
SLC25A34	Down
PRKAA2	Down
BTC	Down
FXYD1	Down
LDB3	Down
ANGPTL7	Down
ATP1A2	Down
CYP11A1	Down
IL36A	Down
CSRNP1	Down
SFTA2	Down
LPIN1	Down
SH3BGR	Down
TGFBR3	Down
SPINK5	Down

IKZF2	Down
ATP2A1	Down
ST6GALNAC1	Down
MLIP	Down
MYOZ3	Down
NCMAP	Down
MYL3	Down
PADI2	Down
GNG7	Down
HP	Down
GGT6	Down
ID4	Down
CLDN8	Down
SCGB2A1	Down
CLDN11	Down
PPP1R1B	Down
SYNGR1	Down
CRACDL	Down
CCDC60	Down
PPL	Down
ACACB	Down
SLC25A4	Down
RBM47	Down
CLIC5	Down
PPP1R12B	Down
PLEKHB1	Down
ACADSB	Down
CLDN10	Down
CAPN3	Down
LEXM	Down
KRT13	Down
SLC9A4	Down
CA13	Down
EXPH5	Down
SLC44A3	Down
FITM1	Down
SORT1	Down
SERPINA5	Down
TMPRSS2	Down
KRT3	Down
TFF3	Down
GAS7	Down

ENPP4	Down
TTC9	Down
AADAC	Down
HSPB8	Down
MYOZ1	Down
CALML6	Down
GULP1	Down
KLHL38	Down
CPEB4	Down
GFRA1	Down
HHATL	Down
SERPINB11	Down
THSD4	Down
IL33	Down
KRT76	Down
TRNP1	Down
PSCA	Down
MACC1	Down
GABRP	Down
LMOD1	Down
HJV	Down
PHYHIP	Down
COQ8A	Down
PLN	Down
VWA5A	Down
ACER1	Down
ACP3	Down
VSIG10L	Down
BICDL2	Down
EYA2	Down
PPM1L	Down
MB	Down
HS3ST1	Down
PRR15L	Down
CMA1	Down
MYH7	Down
ANXA1	Down
MYH6	Down
OCLN	Down
CMYA5	Down
OGN	Down
LMO7	Down

BEX4	Down
FUT3	Down
TMEM125	Down
ADHFE1	Down
TP53INP2	Down
SMYD1	Down
SLC25A23	Down
MPZ	Down
PKIA	Down
PHKG1	Down
KLHDC8A	Down
DUSP13	Down
CH25H	Down
SCEL	Down
PROM1	Down
LDLRAD1	Down
GALNT12	Down
GATM	Down
EHF	Down
DMBT1	Down
TMEM52	Down
UCP3	Down
MYBPC1	Down
ADGRF1	Down
SMTNL1	Down
PIGR	Down
SYPL2	Down
CST4	Down
ICA1	Down
SMTNL2	Down
CEACAM6	Down
DEGS2	Down
CXCL17	Down
WIF1	Down
ADPRHL1	Down
SCN4A	Down
MYOM2	Down
DUSP29	Down
MUSTN1	Down
LIMCH1	Down
VGLL2	Down
SCNN1A	Down

KLHL40	Down
ENO3	Down
CLDN23	Down
SLC1A1	Down
OXGR1	Down
CD24	Down
BSPRY	Down
ABLIM1	Down
SVIP	Down
AZGP1	Down
ALDH1A1	Down
TMC5	Down
PLEKHA7	Down
CAMK2B	Down
FNDC4	Down
ADH1C	Down
KRT23	Down
MYH2	Down
SLC37A1	Down
CACNG6	Down
MPP7	Down
CRYAB	Down
BPIFB2	Down
MANSC1	Down
FGF7	Down
ZFP36	Down
SLC25A25	Down
ZSCAN18	Down
TPRG1	Down
ABI3BP	Down
C4orf54	Down
SCGB1A1	Down
SFRP1	Down
RNF225	Down
INSYN1	Down
MYADML2	Down
TRIM55	Down
NEBL	Down
LNX1	Down
AGR3	Down
SPRR3	Down
EPS8L1	Down

NRAP	Down
USP2	Down
TCEA3	Down
TXLNB	Down
TRDN	Down
MAOB	Down
FOLR1	Down
SPTSSB	Down
ELF5	Down
CYP2E1	Down
IL34	Down
EIF4EBP3	Down
C4orf19	Down
SLC5A1	Down
FOS	Down
MAMDC2	Down
COX6A2	Down
CHL1	Down
CCL28	Down
CACNA1S	Down
DDIT4L	Down
TTN	Down
RP11-467J12.4	Down
TNNC2	Down
CDO1	Down
MYO5B	Down
FHL1	Down
OBSCN	Down
KBTBD12	Down
SCGB3A1	Down
LMOD3	Down
CGN	Down
MYOT	Down
LMOD2	Down
C10orf71	Down
SMARCD3	Down
BLNK	Down
TSPAN12	Down
FUT2	Down
ALOX12	Down
CILP	Down
SPINK7	Down

SPDEF	Down
APOBEC2	Down
TMEM38A	Down
VWA2	Down
PITX1	Down
DUSP1	Down
XIRP2	Down
EGR1	Down
CLDN17	Down
SGCG	Down
DNASE1L3	Down
TGM5	Down
TESC	Down
SERPINB1	Down
SRL	Down
RNF208	Down
ALPK3	Down
MYO5C	Down
CAND2	Down
CYSRT1	Down
MUC5B	Down
CYP4X1	Down
MYH11	Down
LRRC39	Down
DUSP26	Down
SOD3	Down
SYT8	Down
MLPH	Down
BPIFA1	Down
PLEKHS1	Down
RASAL1	Down
BPIFB1	Down
SCGB3A2	Down
TCN1	Down
UGT1A8	Down
STX19	Down
PTN	Down
PTGDS	Down
KLHL41	Down
MUC5AC	Down
CLDN4	Down
DEFB1	Down

CEACAM5	Down
TFF1	Down
LRG1	Down
RNF150	Down
RHCG	Down
TCAP	Down
ZBTB7C	Down
NOS1	Down
CKM	Down
GALNT5	Down
CHPT1	Down
SGCA	Down
PHYHD1	Down
CLEC10A	Down
TMPRSS11E	Down
CCL2	Down
TNNI2	Down
TMEM45B	Down
KIT	Down
RAB11FIP1	Down
SYNPO2L	Down
LYVE1	Down
ATP10B	Down
MYOZ2	Down
ATP6V1C2	Down
RNF222	Down
SLURP1	Down
KLK12	Down
BNIPL	Down
TNNC1	Down
ACTA1	Down
CRAT	Down
MUC20	Down
SLPI	Down
KLK13	Down
PBX1	Down
KLK11	Down
MYPN	Down
PLAAT1	Down
RAET1E	Down
TMOD1	Down
ANKRD2	Down

RGMA	Down
SMPX	Down
CYP2C18	Down
MFAP4	Down
AQP3	Down
ACTN2	Down
TMPRSS11A	Down
DBNDD1	Down
DIO2	Down
HSPB7	Down
TNFRSF19	Down
MYL2	Down
FABP3	Down
SCARA3	Down
RPL3L	Down
CHI3L2	Down
TNS1	Down
NEB	Down
LYZ	Down
PDZRN3	Down
C1orf116	Down
UGT1A7	Down
EEF1A2	Down
SYNM	Down
HRC	Down
RBM24	Down
FOSB	Down
STYXL2	Down
GAMT	Down
STEAP4	Down
SLC7A4	Down
UNC45B	Down
MGP	Down
FOXA1	Down
ODAM	Down
DPT	Down
MYLK2	Down
LCN2	Down
TRIM63	Down
NCCRP1	Down
LYPD2	Down
CLIC6	Down

GSTA1	Down
CSTB	Down
CXCL12	Down
JPH2	Down
MYL1	Down
KRT84	Down
MYH14	Down
CAV3	Down
AGT	Down
SLC34A2	Down
MGST1	Down
PRUNE2	Down
CLIC3	Down
RBP7	Down
NYNRIN	Down
PRSS3	Down
RBP4	Down
MYLPF	Down
MT1M	Down
ACHE	Down
SPTB	Down
SLN	Down
PCP4L1	Down
PLAAT3	Down
NKX2-3	Down
LGR6	Down
TENT5B	Down
SLC16A9	Down
PAX1	Down
ECHDC3	Down
NR4A1	Down
ASB5	Down
MYO18B	Down
MYF6	Down
POF1B	Down
SYNPO2	Down
MUC4	Down
ALDH3A1	Down
SCNN1G	Down
SLC7A2	Down
LDOC1	Down
PRELP	Down

TNNT3	Down
ABO	Down
CYP4F22	Down
EPHB6	Down
GLYATL2	Down
MYBPC2	Down
NMRK2	Down
LIPH	Down
EPHX3	Down
ELF3	Down
NPPC	Down
FCGBP	Down
DES	Down
CP	Down
KRT7	Down
GBP6	Down
STAC3	Down
IP6K3	Down
SUSD4	Down
ELAPOR1	Down
OTOP3	Down
XIRP1	Down
SULT2B1	Down
DHRS9	Down
CD207	Down
WFDC2	Down
CSRP3	Down
CACNG1	Down
TGM1	Down
DAPL1	Down
ADH7	Down
TRIM54	Down
LYNX1	Down
CRCT1	Down
CWH43	Down
IL1RN	Down
SELENOP	Down
TNNI1	Down
PLAC8	Down
CLDN7	Down
ATP12A	Down
CNFN	Down

A2ML1	Down
IGFBP5	Down
RPTN	Down
VSIG8	Down
SERPINB2	Down
PDLIM3	Down
FLNC	Down
PPP1R27	Down
SLC44A4	Down
KRT24	Down
CASQ2	Down
CES1	Down
AGR2	Down
CLDN3	Down
MSMB	Down
FDCSP	Down
ANKRD1	Down
MYH1	Down
FAM25A	Down

Table S2 The expression levels of these 120 HNSCC ERGs.

Gene symbol	logFC	P.Value
LGALS3BP	1.23213794	1.93302E-20
AGRN	1.93103992	1.6105E-34
FN1	3.08916877	2.78956E-19
COL17A1	1.35506532	3.75967E-06
C1S	1.16535748	6.26498E-10
TGFBI	3.29741249	8.56879E-33
TINAGL1	1.56224119	3.50804E-12
LAMB3	2.06761321	5.73063E-24
LAMA3	2.58771735	8.28169E-23
LAMC1	1.4167026	1.89319E-18
LAMC2	3.78415862	7.44816E-39
GPC1	1.01606656	2.24855E-12
LAMB1	1.67594433	3.0409E-24

LAMA5	1.10616833	1.58378E-16
HSP90B1	1.11835045	1.73482E-29
PTGFRN	1.48568802	2.39795E-26
SERPINH1	2.48454891	4.14602E-43
PSMB9	1.67139354	2.34869E-17
ACTN1	1.16748211	4.79153E-12
PLOD1	1.80222774	2.83787E-37
COL12A1	2.96144312	1.62432E-30
CLSTN1	1.10166464	1.90502E-17
TUBA1B	1.03038566	1.48062E-13
LCN2	-1.8664542	2.47178E-07
COL7A1	2.64776421	7.07121E-44
FLNA	1.34789633	2.88999E-18
EXT2	1.03596739	1.67881E-16
ITGA6	2.04036772	9.767E-23
KRT9	1.14409898	4.72063E-09
SERPINE2	1.33966502	9.2794E-08
CD109	1.36459169	3.08496E-16
ANXA1	-1.4977862	3.01762E-13
ITGB4	1.60396463	1.36348E-19
TUBB	1.19991148	1.15137E-31
LOXL2	3.03854026	1.68816E-40
TENM3	1.51035907	7.33325E-13
INHBA	3.5996156	1.26389E-32
PLOD3	1.94016439	4.50614E-35
DNAJB11	1.20228694	1.52577E-28
SLC7A5	1.61269515	2.16591E-15
TNC	2.19189485	9.08128E-14
MSN	1.50105708	1.04524E-22
CTSC	1.52325691	6.05133E-23
ACTN2	-2.429468	4.2388E-08
SFN	1.0333901	1.63782E-07
PYGL	1.28297662	3.61586E-11
EXT1	1.61395017	9.14784E-35
CFL1	1.01759238	5.06958E-35
COL4A2	2.85205794	1.29301E-44
SLC44A1	1.15381128	5.19077E-21
PTPRK	1.14584688	1.77085E-15
KRT5	1.08095279	1.68051E-07
SLC1A5	1.00785194	2.71734E-18
BMP1	1.95805391	2.45777E-45
PFN1	1.02099635	5.32433E-31

MET	1.43038339	9.9176E-17
FSCN1	2.23657094	1.16169E-45
KRT3	-1.9595339	1.32225E-14
RUVBL1	1.21046322	2.108E-31
ARPC1B	1.66816461	8.64513E-38
HLA-A	1.32605813	2.55057E-18
TMEM132A	1.99828608	1.00761E-38
PLOD2	1.5435176	3.3347E-15
KRT75	1.69918407	2.26842E-05
EPCAM	1.36015996	4.2593E-06
SLC16A1	1.77820536	1.34305E-29
HLA-B	1.46199577	2.04702E-17
KRT17	2.68758597	1.28068E-23
ITGAV	1.21374181	1.33389E-11
GMPS	1.12564804	5.61265E-20
ITGA5	2.50669811	4.88174E-30
C1QTNF6	2.59911337	1.25947E-41
FBXO2	1.07784756	3.16511E-05
RCC2	1.16822579	3.60761E-31
KRT14	1.40703629	0.000132412
DSG2	1.48432309	2.29952E-12
ACOT7	1.53890864	4.90032E-41
ATP1B3	1.37869099	1.19323E-24
TSPAN9	1.06550484	1.89174E-17
PFDN2	1.03862269	2.89741E-26
ACTL6A	1.38444783	1.80356E-21
IGSF8	1.03501034	1.80311E-24
SERPINE1	3.28410144	3.97193E-29
FTL	1.2071858	1.57368E-15
TGFB1	1.96973665	4.79779E-57
COL6A1	2.40939441	6.28432E-23
ICAM1	1.42458793	4.93196E-11
IGSF3	1.16469166	2.63746E-14
PCNA	1.24074665	1.2153E-21
AP2M1	1.14358378	1.52003E-28
XYLT2	1.15543142	1.92541E-24
COL4A1	3.0876135	9.00132E-46
MYO1B	1.95666928	1.41403E-28
PDIA4	1.35335452	3.23186E-28
EDIL3	1.04213835	2.25689E-05
ECE1	1.15530596	2.6806E-12
SNRPB	1.06962731	1.5798E-28

TTYH3	2.09702455	2.84682E-38
TAGLN2	1.08685764	1.17569E-36
TPBG	1.74749644	3.47104E-40
LAMA4	1.33780426	2.01732E-12
MCM2	1.98542929	5.21249E-30
THSD4	-1.2531578	4.10517E-14
PLAU	2.88803932	1.08859E-39
CAD	1.06440795	2.37766E-17
HPRT1	1.21663214	3.76021E-29
TSPAN15	1.0531248	2.50782E-12
PIP	-4.2077047	1.33204E-30
MARCKSL1	1.74994586	3.92899E-28
QSOX2	1.34748147	2.77609E-29
EXOSC4	1.00705432	1.09417E-18
SLC16A3	1.30591197	5.16328E-17
POLR2H	1.17368277	8.30718E-22
FUCA2	1.09990131	2.02622E-26
MAMDC2	-1.3151994	9.97573E-11
NASP	1.04968358	6.55608E-20
AZGP1	-2.5369803	1.12609E-11
KYNU	1.16039194	4.19718E-08
PML	1.0960355	3.83435E-17
CASK	1.0475152	4.54615E-15
